# Claudin-low breast cancers: clinical, pathological, molecular and prognostic characterization

**DOI:** 10.1186/1476-4598-13-228

**Published:** 2014-10-02

**Authors:** Renaud Sabatier, Pascal Finetti, Arnaud Guille, José Adelaide, Max Chaffanet, Patrice Viens, Daniel Birnbaum, François Bertucci

**Affiliations:** Department of Molecular Oncology, Centre de Recherche en Cancérologie de Marseille, UMR1068 Inserm, Institut Paoli-Calmettes (IPC), Marseille, France; Department of Medical Oncology, Institut Paoli-Calmettes (IPC), Marseille, France; Faculté de Médecine, Université Aix-Marseille, Marseille, France

**Keywords:** Breast cancer, Claudin-low, Molecular profiling, Prognosis, Response to chemotherapy

## Abstract

**Background:**

The lastly identified claudin-low (CL) subtype of breast cancer (BC) remains poorly described as compared to the other molecular subtypes. We provide a comprehensive characterization of the largest series of CL samples reported so far.

**Methods:**

From a data set of 5447 invasive BC profiled using DNA microarrays, we identified 673 CL samples (12,4%) that we describe comparatively to the other molecular subtypes at several levels: clinicopathological, genomic, transcriptional, survival, and response to chemotherapy.

**Results:**

CL samples display profiles different from other subtypes. For example, they differ from basal tumors regarding the hormone receptor status, with a lower frequency of triple negative (TN) tumors (52% *vs* 76% for basal cases). Like basal tumors, they show high genomic instability with many gains and losses. At the transcriptional level, CL tumors are the most undifferentiated tumors along the mammary epithelial hierarchy. Compared to basal tumors, they show enrichment for epithelial-to-mesenchymal transition markers, immune response genes, and cancer stem cell–like features, and higher activity of estrogen receptor (ER), progesterone receptor (PR), EGFR, SRC and TGFβ pathways, but lower activity of MYC and PI3K pathways. The 5-year disease-free survival of CL cases (67%) and the rate of pathological complete response (pCR) to primary chemotherapy (32%) are close to those of poor-prognosis and good responder subtypes (basal and ERBB2-enriched). However, the prognostic features of CL tumors are closer to those observed in the whole BC series and in the luminal A subtype, including proliferation-related gene expression signatures (GES). Immunity-related GES valuable in basal breast cancers are not significant in CL tumors. By contrast, the GES predictive for pCR in CL tumors resemble more to those of basal and HER2-enriched tumors than to those of luminal A tumors.

**Conclusions:**

Many differences exist between CL and the other subtypes, notably basal. An unexpected finding concerns the relatively high numbers of ER-positive and non-TN tumors within CL subtype, suggesting a larger heterogeneity than in basal and luminal A subtypes.

**Electronic supplementary material:**

The online version of this article (doi:10.1186/1476-4598-13-228) contains supplementary material, which is available to authorized users.

## Background

Breast cancer (BC) is a heterogeneous disease with several classification systems [1]. Molecular classification, based on gene expression profiling, has been a major improvement of BC approach for a decade [2, 3], with the description of five major subtypes associated with different molecular alterations and distinct clinical outcome including therapeutic response: luminal A, luminal B, ERBB2-enriched, basal and normal-like [2, 4].

Following this discovery, additional subgroups of BC were identified such as the interferon-enriched [5] and the molecular apocrine [6] subgroups and several subgroups of triple-negative BCs [7]. In 2007, a new intrinsic subtype was described, the claudin-low subtype (CL), through the combined analysis of murine mammary carcinoma models and human BCs [8]. This subtype represented 6% of the BC samples analyzed (13/232). Surprisingly, since then, only one study focused on the phenotypic and molecular characterization of CL BCs in a series of 76 and 32 cases, respectively [9]. CL tumors lacked tight junction proteins including claudin 3 and E-cadherin, and were characterized by a low expression of luminal markers and a high expression of mesenchymal markers. Enriched in gene expression signatures (GES) derived from human tumor-initiating cells (TICs) and mammary stem cells [8], CL tumors displayed the least differentiated phenotype along the mammary epithelial differentiation hierarchy [9] and were frequent in the residual mammary tumor tissue after either hormone therapy or chemotherapy [10]. Today, with less than 90 samples characterized, the CL subtype is the least characterized subtype in the literature.

We analyzed more than 30 data sets containing almost 5500 clinically annotated BCs profiled using whole-genome DNA microarrays and identified 673 CL samples. We provide here a comprehensive characterization of CL BCs at multiple levels: clinicopathological, genomic (DNA copy number and mutations), transcriptional, survival, response to chemotherapy, and analysis of prognostic and predictive parameters.

## Methods

### Selection of the patients

We collected 32 retrospective data sets of BC samples profiled using oligonucleotide microarrays (Additional file 1: Table S1), including our own set (IPC set) and 31 public sets [3, 6, 9, 11–39]. Regarding our own set, each patient had given written informed consent and the study had been approved by our institutional ethics committee. Gene expression and clinicopathological data of public series were retrieved from NCBI GEO and Array Express databases and authors’ websites. The 32 data sets included a total of 5447 pre-treatment samples of invasive adenocarcinoma.

### Gene expression data pre-processing

Before analysis, we mapped hybridization probes across the two technological oligonucleotide-based platforms (Agilent and Affymetrix) used in these series. Affymetrix gene chips annotations were updated using NetAffx Annotation files (http://www.affymetrix.com; release from 01/12/2008). Agilent gene chips annotations were retrieved and updated using both SOURCE (http://smd.stanford.edu/cgi-bin/source/sourceSearch) and EntrezGene (*Homo sapiens* gene information db, release from 09/12/2008, http://www.ncbi.nlm.nih.gov/gene/). All probes were thus mapped based on their EntrezGeneID. When multiple probes were mapped to the same GeneID, the one with the highest variance in a particular dataset was selected to represent the GeneID.

Data sets were then processed separately as follows. For the Agilent-based sets, we applied quantile normalization to available processed data. For the Affymetrix-based data sets, we used Robust Multichip Average (RMA) [40] with the non-parametric quantile algorithm as normalization parameter. RMA was applied to the raw data from the other series and the IPC series. Quantile normalization or RMA was done in R using Bioconductor and associated packages.

### Gene expression data analysis

To avoid biases related to immunohistochemistry (IHC) analyses across different institutions and to increase the amount of available data, estrogen receptor (ER), progesterone receptor (PR) and ERBB2 expression analyses were done at the mRNA level using gene expression data of their respective gene, *ESR1, PGR* and *ERBB2*. Because *ESR1, PGR* and *ERBB2* expression profiles had bimodal distribution, we identified a threshold of positivity, common to all sets, for each of these genes. Cases with gene expression higher than this threshold were classified as positive; the others were classified as negative [7].

Within each data set separately, the molecular subtypes related to the intrinsic BC classification were determined using the PAM50 classifier [41]. We first identified the genes common between the 50-gene classifier and each expression data set. Next, we used the expression centroid of each subtype as defined by Parker and colleagues [41] and measured the correlation of each sample with each centroid. The sample was attributed the subtype corresponding to the nearest centroid. To be comparable across data sets and to exclude biases resulting from population heterogeneity, expression data were standardized within each data set. To identify CL samples, we used the method described by Prat and colleagues [9]. Briefly, we used the 808 genes from the nine-cell line CL predictor to define the previously described “CL centroid” and “non-CL centroid”, then calculated the Euclidean distance between each sample and each centroid, and assigned the class of the nearest centroid. For non-CL cases, we kept the subtype defined by the PAM50 classifier. To compare the molecular characteristics of CL BCs to those of the other subtypes, we used metagenes and gene signatures associated with different biological processes and pathways. We compared their expression in CL tumors to that in the five other molecular subtypes. We first developed, using an unsupervised approach, two metagenes associated with the luminal and proliferation patterns. They were established from the luminal and proliferation gene clusters identified in the whole-genome hierarchical clustering of 353 IPC samples: genes belonging to these clusters had a correlation rate above 0.75 and the two metagenes corresponded to the mean expression of all genes included in each cluster. We also studied metagenes associated with different immune populations [42]. Epithelial-to-mesenchymal transition (EMT) was analyzed with a core-EMT GES [43] from which we developed a core-EMT metagene defined as the Taube’s Up/Down metagenes ratio. We also focused on previously published GES of pathway activity [44]. Finally, because CL BCs were described as having stem cell features, we applied a differentiation predictor [9] derived from the gene expression profiles of three mammary cell populations: mammary stem cells, luminal progenitors and mature luminal cells [10, 45]

We also tested the prognostic value of previously reported classifiers associated with survival in BC: the 70-gene GES [11], the Genomic Grade Index (GGI) [14], the Recurrence Score (RS) [46], the Risk of Relapse (ROR) score [41], and the stroma-derived GES (B-cell cluster) [47]. We also looked at the prognostic value of signatures identified in ER-negative, triple negative or basal BCs: the kinase immune metagene [48], the LCK metagene [49], the immune response metagene [50]. Out of these 8 prognosis signatures, 4 are rather related to cell proliferation [11, 14, 41, 46] and 4 to immunity [47–50]. Finally, we tested the predictive value of 4 multigene signatures associated with pathological complete response (pCR) after primary chemotherapy in BC: Diagonal Linear Discriminant Analysis–30 predictor (DLDA30) [18], A-score [21], stromal metagene [51], and RB-loss signature [52].

### Array-comparative genomic hybridization

We compared the genomic profile of CL tumors with that of the other molecular subtypes by analyzing our array-comparative genomic hybridization (aCGH) database containing 256 BCs [53]. Data had been generated by array-comparative genomic hybridization (aCGH) using 244 K CGH Microarrays (Hu-244A, Agilent Technologies). Data analysis was done as previously described [53]. Extraction of data (log2 ratio) was done from CGH Analytics, whereas normalized and filtered log2 ratio was obtained from “Feature Extraction” software (Agilent Technologies). Frequencies of copy number alterations of CL tumors were compared to that of all other breast tumors using Fisher’s exact test with a 5% level of significance. To identify chromosomal regions with a statistically high frequency of copy number alterations (CNAs), we used the GISTIC algorithm [54]. The altered genes were compared to those described in CL cases from a mouse model of P53null tumors [55]. We also determined the genomic patterns of tumors using Hicks’ classification [56].

### Statistical analysis

Correlations between sample groups and clinicopathological features were calculated with the Fisher’s exact test or the Student’s *t*-test when appropriate. Disease-free survival (DFS) was calculated from the date of diagnosis to the date of first event (loco-regional or metastatic relapse, death), and follow-up was measured to the date of last news for event-free patients. Breast cancer patients with metastasis at diagnosis were excluded from DFS analysis. Survival curves were obtained using the Kaplan-Meier method and compared with the log-rank test. Prognostic analyses used the Cox regression method. Univariate analyses tested classical clinicopathological features: age, pathological tumor size (pT ≤ 20 mm *vs* >20), axillary lymph node involvement (pN positive *vs* negative), SBR grade (1 *vs* 2–3), *ESR1, PGR* and *ERBB2* status (negative *versus* positive), triple-negative status (yes *versus* no), and pathological subtype. We also analyzed the pathological response after neoadjuvant treatment which was available in 6 public sets [18, 19, 23, 25, 34, 39]. All statistical tests were two-sided at the 5% level of significance. Analyses were done using the survival package (version 2.30), in the R software (version 2.15.2). Our analysis adhered to the REporting recommendations for tumor MARKer prognostic studies (REMARK) [57]. A Sweave report describing the analysis of gene expression data and the associated statistical analysis has been generated and is available as Additional file 2.

## Results

### Molecular subtypes

We collected public gene expression and clinicopathological data of a total of 5447 distinct invasive breast carcinomas. We determined the molecular subtype of tumors in each data set separately by using the PAM50 classifier [41] and the claudin-low predictor [9]: 1494 samples were luminal A (27.4%), 1077 (19.8%) were luminal B, 749 (13.8%) were ERBB2-enriched, 1003 (18.4%) were basal, 451 (8.2%) normal-like, and 673 (12.4%) were CL. Seventy-eight percent of CL cases identified were initially attributed by the PAM50 classifier to the basal (53%) and normal-like (25%) subtypes. Only 11% were luminal A, 7% ERBB2-enriched and 4% luminal B.

For validation of the claudin-low predictor that we applied, we compared our findings with those described by Prat and colleagues in three data sets common with ours [9, 11, 18] and found 98.5% of concordant classification (Cl vs non-CL) out of the 337 tested samples (332 samples accurately classified), with a specificity of our predictor equal to 100% (all 32 CL samples according to our predictor were CL according to Prat’s predictor) and a sensitivity equal to 86% (5 out of 305 non-CL samples according to our predictor were CL according to Prat’s predictor).

### Clinicopathological characteristics

Results, both descriptive and comparative, are shown in Table 1. Each variable was compared between the CL subtype and each of the other subtypes. Forty-nine percent of patients with CL tumor were 50-year old or younger. Patients with CL tumor were younger than those with luminal A, ERBB2-enriched or luminal B tumors, and older than patients with basal tumors. Most CL cases were ductal carcinomas (78%). Other histological types included lobular carcinomas (4%), carcinomas of mixed histology (4%), and medullary carcinoma (3%). As expected, most of the metaplastic carcinomas were CL (5 out of 7: 71%). Histological grade of CL tumors was often high (grade 3: 56%) or intermediate (grade 2: 35%), with grade 1 observed in only 9% of cases. Differences with the other subtypes were very significant with the basal subtype, which contained more grade 3 samples, and with the luminal A subtype, which contained less grade 3, and significant but to a lesser extent with the three other subtypes (intermediate between ERBB2-enriched and luminal B subtypes).

**Table 1:** **Clinicopathological characteristics of invasive breast cancers according to the molecular subtypes**

Variables	N	Claudin-low	Luminal A	Basal	ERBB2-enriched	Luminal B	Normal-like
	5447	673	1494	1003	749	1077	451
Age at diagnosis, years							
<=50	1834	238(49%)	470(43%)	423(56%)	224(43%)*	328(46%)*	151(53%)*
>50	2005	247(51%)	622(57%)	331(44%)	291(57%)	380(54%)	134(47%)
Histological type							
IDC	1181	140(78%)	263(76%)	224(88%)	201(89%)	255(89%)	98(84%)
ILC	72	8(4%)	34(10%)	4(2%)	4(2%)	12(4%)	10(9%)
MED	24	5(3%)	1(0%)	18(7%)	0(0%)	0(0%)	0(0%)
MIX	59	7(4%)	23(7%)	4(2%)	10(4%)	12(4%)	3(3%)
other	77	20(11%)	26(7%)	6(2%)	11(5%)	8(3%)	6(5%)
Histological grade							
1	489	49(9%)	293(26%)	12(2%)	15(3%)	53(7%)	67(21%)
2	1579	180(35%)	605(54%)	104(14%)	170(32%)	367(47%)	153(48%)
3	1957	290(56%)	222(20%)	640(85%)	350(65%)	358(46%)	97(31%)
Pathological tumor size							
pT1	928	106(38%)	343(46%)	130(29%)	89(27%)	163(34%)	97(50%)
pT2-3	1542	172(62%)	399(54%)	323(71%)	239(73%)	311(66%)	98(50%)
Pathological axillary lymph node status							
negative	1907	180(54%)	550(61%)	379(65%)	232(51%)*	387(60%)*	179(59%)*
positive	1313	153(46%)	355(39%)	200(35%)	224(49%)	258(40%)	123(41%)
*ESR1* expression status							
negative	1929	433(64%)	80(5%)	859(86%)	437(58%)	24(2%)	96(21%)
positive	3518	240(36%)	1414(95%)	144(14%)	312(42%)	1053(98%)	355(79%)
*ESR1* mRNA expression, median	5447	6.46	10.55	5.46	7.18	10.76	9.77
*PGR*expression status							
negative	2851	445(66%)	386(26%)	864(86%)	567(76%)	443(41%)	146(32%)
positive	2594	228(34%)	1108(74%)	139(14%)	181(24%)	634(59%)	304(68%)
*PGR* mRNA expression, median	5445	4.21	5.24	3.54	4.07	4.52	5.07
*ERBB2* expression status							
negative	4738	646(96%)	1407(94%)*	958(96%)*	320(43%)	1011(94%)*	396(88%)
positive	709	27(4%)	87(6%)	45(4%)	429(57%)	66(6%)	55(12%)
*ERBB2* mRNA expression, median	5447	6.45	7.5	6.52	8.84	7.59	7.83
Triple-negative expression status							
yes	1336	352(52%)	22(1%)	762(76%)	138(18%)	13(1%)	49(11%)
no	4110	321(48%)	1472(99%)	241(24%)	610(82%)	1064(99%)	402(89%)
Pathological complete response							
pCR	302	73(32%)	21(7%)	104(33%)*	56(37%)*	40(18%)	8(14%)
RD	992	155(68%)	302(93%)	210(67%)	97(63%)	178(82%)	50(86%)
DFS event							
no	2190	223(65%)	736(75%)	396(62%)*	222(52%)	395(60%)*	218(73%)*
yes	1165	120(35%)	246(25%)	245(38%)	205(48%)	268(40%)	81(27%)
5-year DFS [95CI]	3355	67% [0.62-0.73]	79% [0.77-0.83]	60% [0.56-0.64]	55% [0.5-0.6]	64% [0.6-0.68]	79% [0.75-0.84]

IDC: invasive ductal carcinoma; ILC: invasise lobular carcinoma; MED: medullary carcinoma; MIX: mixed; pCR: pathological complete response; RD: residual disease; DFS: disease-free survival; OR: odd ratio; 95CI: 95% confidence interval.

*p-value < 0.05.

Thirty-eight percent of CL tumors measured 2 cm or less (pT1), a percentage intermediate between that of highly proliferative subtypes (basal, ERBB2-enriched, and luminal B) and that of less proliferative ones (luminal A and normal-like). Forty-six percent of CL samples presented pathological axillary lymph node involvement at diagnosis. This ratio was significantly lower in basal (35%) and luminal A (40%) samples. Most tumors (77%) with lymph node involvement were larger than 2 cm. However, the positive correlation between pT (pT1 *vs* pT2-3) and the axillary lymph node status (negative *vs* positive) was weaker in CL tumors (OR = 2.58) and basal tumors (OR = 2.20) than in luminal A (OR = 3.60) or normal-like (OR = 6.69) tumors.

Sixty-four percent and 66% of CL samples were classified as negative for *ESR1* and *PGR* respectively. As expected, differences were highly significant when compared with the two luminal and the normal-like subtypes, which were much more frequently positive for *ESR1* and *PGR*. A small difference was observed with the ERBB2-enriched subtype. More unexpected was the strong difference observed with the basal subtype, which contained many more tumors negative for *ESR1* and *PGR*. Ninety-six percent of CL tumors were negative for *ERBB2*, representing the highest percentage among all subtypes. The difference was not significant with the basal subtype, but significant with the ERBB2-enriched and normal-like subtypes. Fifty-two percent of CL tumors were triple negative (TN), significantly less than basal tumors (76%) and more than ERBB2-enriched samples (18%) and luminal A and B samples (1% each). Twenty-seven percent of TN breast cancers (TNBC) belonged to the CL subtype.

### DNA copy number profiles

**Figure 1:**
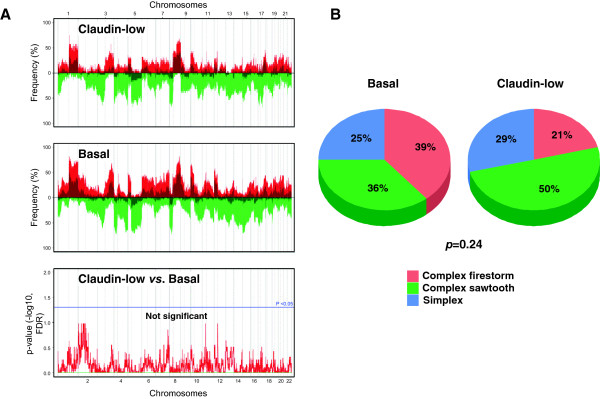
**Comparative genomic analysis of claudin-low and basal breast cancers. A)** Frequency plots of DNA copy number alterations in claudin-low samples (N = 28) and basal samples (N = 61). Frequencies (vertical axis, from 0 to 100%) are plotted as a function of chromosome location (from 1pter on the left to 22qter on the right). Vertical lines indicate chromosome boundaries. Positive and negative values indicate frequencies of tumors showing copy number increase and decrease, respectively, with gains (in red), amplifications (dark red), losses (in green) and deletions (dark green). Bottom: supervised analysis comparing the genomic profiles of CL *versus* basal cases. The difference was assessed with the Fisher’s exact test. The blue line indicates the limit of significance (*p* = 0.05). **B)** Genomic patterns of CL and basal tumors using Hicks’ classification [56]. The difference between the subtypes was assessed with the Pearson's Chi-squared test.

Most of the 28 CL samples profiled using aCGH displayed several gains and losses suggesting a high genomic instability. Because basal tumors are also known to be highly instable, we compared their genomic profile to those of CL samples: no difference could be observed with many gains and losses in both subtypes (Figure 1A). In the same way, supervised analysis of CNAs between CL and non-CL samples did not find any genomic region specifically gained or lost in CL tumors. To identify the most gained or lost regions, we used the GISTIC algorithm. Out of the 10 most gained regions we found 7p11.2 including *EGFR,* 17q12 (*ERBB2*), 17q21.32 (*HOXB* family), 4q13.3 (*CXCL2, 3, 5 and 6*), 11q13-q14 (*PAK1*) and 17q21.33 (*MYST2*, *PDK2*). Some of the most lost regions were 8p23-p12 (*DOK2*, *FGFR1*), 4p16.3 (*SPON2*, *FGFRL1*), 17q21.2-q21.31 (*STAT3*) and 17p13.1-p12 (*TP53, MAP2K4*). Except *TP53*, none of these genes were identified in aCGH analyses performed on P53 null mice tumors [55].

Breast cancers can be classified in three classes according to their genomic patterns [56]. Using this classification, we observed 29%, 21% and 50% of simplex, firestorm and sawtooth CL tumors, respectively. By comparing the genomic patterns between molecular subtypes, we found that CL samples displayed the smallest percentage of firestorm profiles, the largest percentage of sawtooth profiles, and a percentage of simplex profiles intermediate between that of non-aggressive (luminal A and normal-like) and aggressive (basal, ERBB2-enriched and luminal B) subtypes. Based on these percentages, CL tumors were different from ERBB2-enriched tumors (*p* = 4.45 E-04, Fisher’s exact test) and luminal B tumors (*p* = 1.34 E-03) with more complex sawtooth tumors (Additional file 3: Table S2), whereas they were not different from basal BCs (*p* = 0.24; Figure 1B).

### Transcriptional profiles

**Figure 2:**
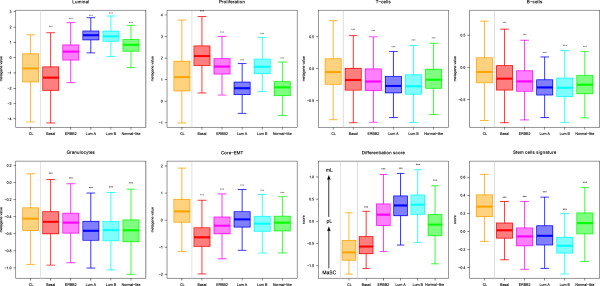
**Comparison of gene expression signatures across molecular subtypes.** Box plots of expression metagenes and scores across molecular subtypes: luminal, proliferation, immune, and core-EMT metagenes, differentiation score (mL, mature luminal; pL, porogenitor luminal; MaSC, mammary stem cells), stem cells score. P-values (*t*-test) of comparisons between CL and each of the other subtypes are shown as follows: *, ≤5%; **, ≤1%; ***, ≤0.1%.

We compared the mRNA expression of different genes and pathways in CL *versus* other subtypes. As expected, CL tumors showed low expression of *ESR1*, *PGR* and *ERBB2* genes (Table 1) and low expression of associated genes as demonstrated by the low expression of the luminal metagene (Figure 2) and the ER, PR and ERBB2 activation pathways signatures (Additional file 4: Figure S1). Regarding these genes and signatures, significant differences existed between CL and the other subtypes, including the basal subtype. CL BCs also differed from basal BCs in other aspects. Expression of the proliferation-related metagene in CL tumors was lower than in basal tumors, but higher than in luminal A and normal-like tumors (Figure 2 and Additional file 5: Table S3). CL tumors displayed lower expression of MYC, PI3K, and β-catenin activation pathways when compared to basal cases, with activity levels close to those of luminal A tumors for MYC and PI3K (Additional file 4: Figure S1). By contrast, they showed higher expression than basal tumors of EGFR, SRC, TGFβ and STAT3 activation pathways. We also analyzed the expression of immune response GES [42]. CL tumors overexpressed T-cells, B-cells and granulocytes metagenes as compared to the other subtypes (Figure 2). They also highly expressed the IFNγ activation pathway with similar level than that of basal cases (Additional file 4: Figure S1).

We then focused on the expression of genes associated with epithelial-to-mesenchymal transition (EMT). As shown in Additional file 6: Figure S2, CL tumors displayed the lowest expression of genes coding for epithelial cell-cell adhesion molecules (*CDH1*, *claudin 3, claudin 4, claudin 7* and *occludin*) and the highest expression of *vimentin, SNAI1* and *2*, *TWIST1* and *2*, and *ZEB1* and *2*, known to be transcriptional repressors of *CDH1*. This EMT pattern was confirmed using a GES associated with EMT [43]: CL tumors had the highest expression of the core-EMT metagene when compared to the other subtypes (Figure 2).

Following the hypothesis that the molecular subtypes are emerging at different stages of mammary cell differentiation [45], we evaluated the differentiation degree of CL tumors. Using a previously published differentiation score [9], we observed that most of the CL cases (96%) presented a score between those of mammary stem cells and those of luminal progenitors (Figure 2). Only 4% had a score close to those of mature luminal cells. This pattern of differentiation was similar, although lightly inferior, in basal tumors (92% between mammary stem cells and luminal progenitors) and very different in the other subtypes. Only 35% of ERBB2-enriched and nearly 15% of luminal samples had a low differentiation score close the stem cell profile.

We then classified all samples according to a GES of breast cancer stem cells (CD44^+^/CD24^-/low^-mammospheres-forming cells) [10]. CL tumors were strongly associated with the signature (Figure 2), suggesting enrichment in stem cell features. Similarly, the expression of gene markers of tumor-initiating cells (*ALDH1A1*, *CD29*, *INPP5D*) was different between the CL subtype and the other subtypes, including the basal subtype (data not shown).

### Disease-free survival and prognostic features

**Figure 3:**
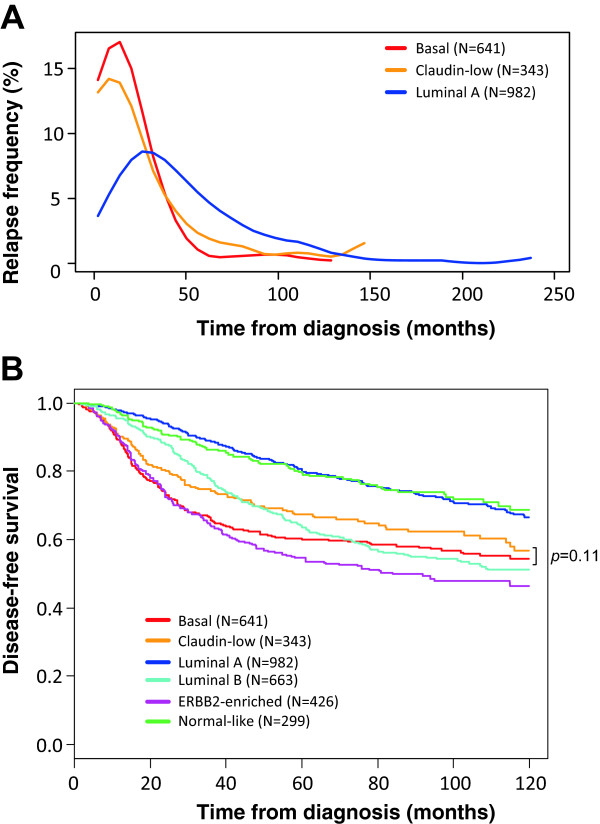
**DFS according to molecular subtypes. A)** Frequencies of relapses according to time from diagnosis between luminal A, basal and CL breast cancers. **B)** Kaplan-Meier DFS curves in the 6 subtypes (*p*-value for comparison between CL and basal tumors is shown, log-rank test).

Clinical outcome was available for 3682 out of 5447 patients with 5-year DFS rate equal to 67% (CI95, 66–69), including 343 out of 673 with CL BC. In the CL subtype, the median follow-up was 72 months for the 251 event-free patients. A total of 130 patients (34%) displayed a DFS event. Similarly to the basal subtype (and differently from the luminal A subtype), most of the relapses occurred in the first three years (Figure 3A), with median times to relapse of 19 months and 17 months for CL and basal tumors, respectively. The 5-year DFS rate was 67% (CI95, 62–73; N = 343) in the CL subtype (Figure 3B), intermediate between that observed in ERBB2-enriched BC patients (55% 5-year DFS, *p* = 2.3 E-03, log-rank test; N = 426) and luminal A BC patients (79% 5-year DFS, *p* = 6.7 E-07, log-rank test; N = 982) and normal-like BC patients (79% 5-year DFS, *p* = 4.7 E-04, log-rank test; N = 299). The prognosis of CL cases was not different from that of luminal B samples (64% 5-year DFS; *p* = 0.56, log-rank test; N = 663), and was better although not significantly different from that of basal tumors (60% 5-year DFS rate; *p* = 0.11, log-rank test; N = 641). Unfortunately, the site of first metastatic relapse was not informed in most of the cases studied.

We then performed prognostic analyses in the CL subtype by assessing the prognostic impact of the usual clinicopathological features. In univariate analysis, the well-known unfavorable clinicopathological features (pT > 2 cm, grade 2–3, pN-positive, low *ESR1* expression, low *PGR* expression, and *ERBB2* overexpression) were associated with shorter DFS in patients with CL tumor (Table 2). Comparison with the results observed in the whole BC series and in each of the other subtype (Additional file 7: Table S4) revealed that the prognostic features were the same in the CL subtype and in the whole series, totally different between the CL and the other proliferative subtypes (basal, ERBB2-enriched and luminal B). The largest similarity was observed with the luminal A subtype.

**Table 2:** **Univariate Cox regression analysis for DFS**

A. Clinicopathological features		Claudin-Low			All samples		
		n	HR [95CI]	***p***-value	n	HR [95CI]	***p***-value
Age at diagnosis, years	>50 *vs* ≤50	237	0.9 [0.57-1.4]	0.636	2366	1.01 [0.87-1.16]	0.923
Histological type	ILC *vs* IDC	59	0.00 [0.00- Inf]	0.837	624	1.09 [0.71-1.67]	0.269
	MED *vs* IDC		0.45 [0.06-3.35]			0.53 [0.20-1.42]	
	MIX *vs* IDC		0.00 [0.00- Inf]			0.44 [0.18-1.06]	
	other *vs* IDC		1.90 [0.44-8.10]			1.04 [0.57-1.92]	
Histological grade	2-3 *vs* 1	270	3.63 [1.32-9.92]	**1.22E-02**	2552	2.36 [1.86-3.01]	**2.84E-12**
Pathological tumor size	pT2-3 *vs* pT1	155	2.33 [1.27-4.26]	**6.01E-03**	1744	1.52 [1.29-1.8]	**6.83E-07**
Pathological axillary lymph node status	positive *vs* negative	194	2.23 [1.34-3.72]	**2.01E-03**	2365	1.45 [1.26-1.68]	**3.95E-07**
*ESR1* expression status	positive *vs* negative	343	0.38 [0.25-0.58]	**5.02E-06**	3355	0.56 [0.5-0.63]	**<2.0E-16**
*PGR* expression status	positive *vs* negative	343	0.56 [0.38-0.83]	**3.86E-03**	3353	0.7 [0.63-0.79]	**2.24E-09**
*ERBB2* expression status	positive *vs* negative	343	1.98 [0.92-4.26]	0.079	3355	1.4 [1.18-1.65]	**7.22E-05**
Triple-negative expression status	yes *vs* no	343	2.19 [1.51-3.18]	**3.82E-05**	3354	1.7 [1.49-1.94]	**1.22E-15**
**B. Gene expression signatures**							
70-gene GES [11]	Poor *vs* Good	343	1.22 [0.74-2.02]	0.435	3355	1.83 [1.57-2.12]	**3.00E-15**
GGI [14]	Poor *vs* Good	283	1.42 [0.92-2.2]	0.113	2655	2.1 [1.82-2.43]	**<2.0E-16**
RS [46]	Intermediary *vs* Good	343	2.73 [1.40-5.31]	**1.09E-03**	3355	1.63 [1.38-1.93]	**<2.0E-16**
	Poor *vs* Good		3.03 [1.68-5.46]			2.15 [1.87-2.47]	
ROR [41]	Intermediary *vs* Good	343	1.28 [0.69-2.36]	**8.74E-03**	3355	1.75 [1.45-2.10]	**<2.0E-16**
	Poor *vs* Good		1.85 [1.24-2.75]			2.11 [1.85-2.41]	
Immune response metagene [50]	Poor *vs* Good	343	1.17 [0.82-1.68]	0.387	3354	1.19 [1.06-1.34]	**2.93E-03**
LCK metagene [49]	Poor vs Good	343	1.12 [0.78-1.6]	0.541	3355	1.1 [0.96-1.26]	0.152
Kinase immune metagene [48]	Poor *vs* Good	343	1.14 [0.78-1.65]	0.507	3355	1.04 [0.88-1.24]	0.615
B-cell cluster [47]	Intermediary *vs* Good	343	1.15 [0.74-1.79]	0.0692	3353	1.20 [1.04-1.38]	**3.94E-05**
	Poor *vs* Good		1.64 [1.07-2.52]			1.38 [1.20-1.59]	

IDC: invasive ductal carcinoma; ILC: invasise lobular carcinoma; MED: medullary carcinoma; MIX: mixed; HR: hazard ratio; 95CI: 95% confidence interval.

P-values < 0.05 are represented in boldface.

We also compared the prognostic value of 8 prognostic GES in the different subtypes (Table 2 and Additional file 7: Table S4). Whereas 6 signatures (4 proliferation-related and 2 immunity-related) showed prognostic value in the whole series of samples, only two conserved their prognostic value in CL tumors (Table 2): the RS (HR = 3 when comparing high risk to low risk cases, *p* = 1.1 E-03) and the ROR (HR = 1.85, p = 8.7E-04). There was a trend for the B-cell cluster (HR = 1.6 when comparing poor *vs* good-prognosis groups cases, *p* = 0.07). The 2 other proliferation-related signatures (70-gene GES and GGI) and the 3 other immunity-related signatures (immune response, LCK, and kinase immune metagenes) had no prognostic value in the CL population. By contrast, the results were very different in the other subtypes (Additional file 7: Table S4). For example, most of the immunity-related signatures were significant in the basal and ERBB2-enriched subtypes, whereas none of the proliferation-related classifiers had a prognostic value in this population in contrast with the luminal A subtype. Results were also different in the luminal B subtype, where 3 proliferation-related and 2 immunity-related signatures showed prognostic value. Altogether, these results suggest that CL tumors have different prognostic features than the other subtypes.

### Pathological response to chemotherapy and predictive features

Pathological response to neoadjuvant chemotherapy was available for 1294 patients out of 5447 patients with a pCR rate equal to 23%. Among the 228 CL samples with data available, the pCR rate was 32% (Table 1), higher than in luminal A (7%, *p* < E-04, Fisher’s exact test; N = 323), luminal B (18%, *p* = 1.1 E-03; N = 218), and normal-like tumors (14%, *p* = 5.4 E-03; N = 58), and similar to the rate observed in basal (33%, *p* = 0.85; N = 314) and ERBB2-enriched cases (37%, *p* = 0.38; N = 153).

Analysis of predictive value of clinicopathological features in CL tumors (Table 3) showed that pCR rates tended to be higher in high grade tumors (*p* = 0.06, Fisher’s exact test) and in samples with low *ESR1* expression (*p* = 0.07). By contrast (Additional file 8: Table S5), *ESR1* expression level did not tend to have predictive value in the basal and ERBB2-enriched subtypes. We also tested the predictive value of 4 GES published as predictive of pathological response in breast cancer treated by anthracycline-based chemotherapy. Only two were associated with pCR in CL tumors: the DLDA30 predictor (*p* = 1.6 E-02, Fisher’s exact test), and the A-score (*p* = 3.2 E-03), which also predicted pCR in the basal and ERBB2-enriched subtypes. By contrast, the stromal metagene and the RB-loss signature failed to predict pCR in CL tumors, whereas they predicted pCR in basal and ERBB2-enriched cancers, respectively. Finally, 3 out of 4 signatures were associated with pCR in the whole series of 1294 samples.

**Table 3:** **Univariate Fisher’s exact test analysis for pathological complete response according to clinicopathological and molecular features**

A. Clinicopathological features	Claudin-low					All samples				
	N	RD	pCR	***p***-value*	OR [95CI]	N	RD	pCR	***p***-value*	OR [95CI]
Age at diagnosis, years				0.392	0.76				0.09	0.8
≤50	123	80 (52%)	43 (59%)		[0.41-1.37]	697	521 (53%)	176 (58%)		[0.61-1.04]
>50	104	74 (48%)	30 (41%)			595	469 (47%)	126 (42%)		
Pathological tumor size				1.00	0.93				0.859	1.1
pT1	5	3 (8%)	2 (9%)		[0.1-11.96]	44	32 (14%)	12 (13%)		[0.53-2.53]
pT2-3	55	34 (92%)	21 (91%)			279	196 (86%)	83 (87%)		
Histological grade				0.06	Inf				**1.68E-04**	8.1
1	8	8 (6%)	0 (0%)		[0.82-Inf]	53	51 (6%)	2 (1%)		[2.11-69.38]
2-3	204	136 (94%)	68 (100%)			1126	854 (94%)	272 (99%)		
Histological type				0.125					0.75	
IDC	77	45 (67%)	32 (84%)			468	337 (82%)	131 (85%)		
ILC	5	3 (4%)	2 (5%)			15	10 (2%)	5 (3%)		
MIX	6	6 (9%)	0 (0%)			37	29 (7%)	8 (5%)		
other	17	13 (19%)	4 (11%)			47	36 (9%)	11 (7%)		
*ESR1* expression status				0.07	0.47				**7.21E-18**	0.3
negative	183	119 (77%)	64 (88%)		[0.19-1.07]	697	470 (47%)	227 (75%)		[0.22-0.4]
positive	45	36 (23%)	9 (12%)			597	522 (53%)	75 (25%)		
*PGR*expression status				0.42	0.64				**4.24E-08**	0.38
negative	195	130 (84%)	65 (89%)		[0.24-1.57]	979	716 (72%)	263 (87%)		[0.26-0.56]
positive	33	25 (16%)	8 (11%)			315	276 (28%)	39 (13%)		
*ERBB2* expression status				0.472	1.7				**5.90E-04**	1.9
negative	219	150 (97%)	69 (95%)		[0.33-8.34]	1125	881 (89%)	244 (81%)		[1.31-2.7]
positive	9	5 (3%)	4 (5%)			169	111 (11%)	58 (19%)		
Triple-negative expression status				0.285	1.4				**8.16E-10**	2.3
no	71	52 (34%)	19 (26%)		[0.74-2.83]	777	642 (65%)	135 (45%)		[1.73-2.97]
yes	157	103 (66%)	54 (74%)			517	350 (35%)	167 (55%)		
DLDA30 [18]				**1.61E-02**	0.49				**7.53E-23**	0.26
pCR-like	120	73 (47%)	47 (64%)		[0.27-0.91]	439	264 (27%)	175 (58%)		[0.2-0.35]
RD-like	108	82 (53%)	26 (36%)			855	728 (73%)	127 (42%)		
Stromal metagene [51]				0.623	1.2				0.12	0.81
pCR-like	55	39 (25%)	16 (22%)		[0.59-2.5]	642	480 (48%)	162 (54%)		[0.62-1.06]
RD-like	173	116 (75%)	57 (78%)			652	512 (52%)	140 (46%)		
A-score [21]				**3.15E-03**	3.9				**1.48E-04**	2
RD-like	37	31 (32%)	6 (11%)		[1.46-12.32]	258	196 (46%)	62 (30%)		[1.36-2.85]
pCR_Like	118	67 (68%)	51 (89%)			378	233 (54%)	145 (70%)		
RB-loss signature [52]				1.00	0.96				**6.52-03**	1.7
Low (RD-like)	212	144 (93%)	68 (93%)		[0.25-3.15]	1150	895 (90%)	255 (84%)		[1.14-2.51]
High (pCR-like)	16	11 (7%)	5 (7%)			144	97 (10%)	47 (16%)		

*, Fisher’s exact test.

IDC: invasive ductal carcinoma; ILC: invasise lobular carcinoma; MIX: mixed; pCR: pathological complete response; RD: residual disease; OR: odds ratio; 95CI: 95% confidence interval.

P-values < 0.05 are represented in boldface.

## Discussion

We provide a comprehensive characterization of a series of 673 CL BCs collected though a meta-analysis of public gene expression data. This represents the largest series reported so far in the literature, with nearly 9-fold more samples than in the pioneering study [9]. We defined the CL breast tumors using the published cell line-based CL predictor [9], which in our hands gave a very high degree of concordance (98.5%) with the predictor originally reported in a common set of 337 samples, suggesting that the CL subtype that we define here overlaps the CL subtype originally described. The subtype of non-CL samples was defined using the classical PAM50 classifier [41]. Using these standard classifiers, we observed the expected incidence of each subtype. The incidence of CL tumors was 12.4%, similar to the 7 to 14% incidence reported by Prat and colleagues in 3 distinct small databases [9]. In our analysis, the PAM50 classifier attributed most of the current CL tumors to the basal and normal-like subtypes (53% and 25%, respectively) as previously described [9]. The large number of samples in each subtype provided an unprecedented opportunity to describe the characteristics of CL tumors and to perform prognostic and predictive analyses specifically in this subtype, comparatively with the other subtypes. Also for the first time, we present genomic data of human CL tumors.

**Figure 4:**
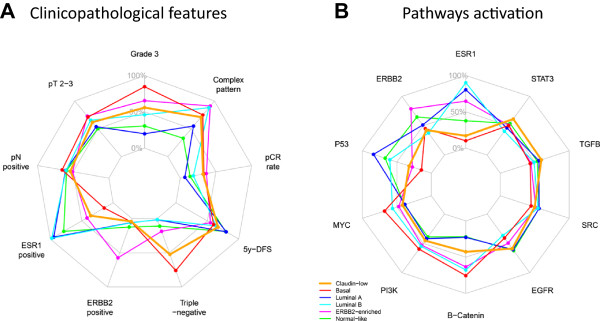
**Radar charts comparing clinicopathological, genomic and transcriptional features through the 6 main molecular subtypes.** Each axis of the diagrams represents a scale of proportions for a specific feature, ranging from 0% to 100%. The proportion of a given feature in a given molecular subtype is reported on the corresponding axis. **A)** Clinicopathological features. **B)** Probabilities of pathway activation calculated according to [44].

Only one published study [9] has described so far the clinicopathological characteristics of CL samples, but information was relatively limited: pathological size, grade, axillary lymph node status and IHC ER status were available for 76 cases, and PR and ERBB2 status for 55 cases. Our percentages of CL tumors with pT2-T3 size (62%), with pN- status (54%) and with grade 3 (56%) are close to those reported by Prat (65%, 47% and 62% respectively). Differences are more important and thus unexpected regarding the hormone receptors and ERBB2 status. In Prat’s study, 79%, 77% and 84% of CL samples were IHC ER-negative (out of 71 informative samples tested at the protein level with IHC), PR-negative (out of 40 informative samples) and ERBB2-negative (out of 45 informative samples) respectively, *versus* 64%, 66% and 96% in our transcriptional analysis, respectively (Figure 4). Similarly, the percentage of IHC TN samples was 67% in Prat’s study (out of 39 informative samples) *versus* 52% in ours (out of 673 samples tested at the mRNA level with DNA microarrays). These discordances may be due to various reasons. The first one may be the difference of technology used to define the ER, PR, ERBB2 and TN status (IHC *versus* mRNA expression profile), even if differences are known to be limited [58]. Thanks to the simultaneous availability of IHC ER, PR and ERBB2 status for 2259 breast cancer samples of our pooled series, including 294 CL samples, we could redefine the TN status at the protein level as did Prat and colleagues. We found results similar or very close to those observed at the mRNA level in the whole series of 673 samples: 52% of CL samples were TN, 63% were ER-negative, 68% were PR-negative and 89% were ERBB2-negative, *versus* 52% 64%, 66% and 96% respectively in our transcriptional analysis. Of note, the results remained exactly the same after exclusion of the Prat’s samples. The second and likely main reason for discrepancy lies in the large quantitative difference in series analyzed: we defined the ER and TN status of CL samples in a series of 673 samples, whereas Prat et al. defined the ER status on three small series of 32 (UNC337), 21 samples (NKI295) and 18 samples (MDACC), and the TN status on two small series of 21 (UNC337) and 18 samples (MDACC) with relative large variations across series regarding the percentage of ER-negative cases (from 67 to 88%) and TN cases (from 61 to 71%). Prat and colleagues did not compare statistically the clinicopathological features of the CL subtype with those of the other subtypes, likely because of the series size limitation. In our analysis, CL BCs displayed only one feature common with basal tumors (*ERBB2* status), whereas differences were significant regarding all the other features: age at diagnosis (less young patients in CL cases), pathological type (less often ductal or medullary, but more often metaplastic in CL), grade (less often grade 3 in CL), tumor size (less often pT2-3 in CL), lymph node status (more often positive in CL), ER and PR status (less often negative in CL), and triple-negative status (less frequent in CL).

To our knowledge, the genomic profiles of CL tumors have been reported only in two studies that described the CNA patterns of a total of 5 CL murine p53-null tumors [8, 55]. Based on the analysis of 28 samples, we showed that CL tumors have a high genomic instability with many gains and losses, frequent complex sawtooth patterns, and the smallest percentage of firestorm profiles. This profile is close to what has been already published concerning basal cases [53], and no significant difference could be identified with basal tumors. It is of note that the main regions we identified as gained or lost in CL samples were not described in genomic analyses of previously published murine models [55]. This suggests that, like for the basal subtype, CL tumorigenesis may be driven by several oncogenic events, and not by a single driver as it can be observed in ERBB2-enriched tumors [59].

At the transcriptional level, we found that the CL subtype differs from the other subtypes in many aspects (Figure 4). We confirmed that CL tumors lack luminal differentiation markers, and show enrichment for EMT markers, immune response genes, and cancer stem cell–like features. They also have a relative low expression of the P53 pathway suggesting apoptosis inhibition. They differ from basal BC at several levels. Both have lower expression of *ESR1*, *PGR*, *ERBB2* genes and ER, PR and ERBB2 activation pathways than the other subtypes, but the expression of ER and PR genes and pathways is higher in CL tumors than in basal tumors. CL BCs are also less proliferative than basal cancers. They overexpress genes associated with immune response and stroma and have higher expression of EMT-related genes and signatures, thus confirming previous results. We also confirmed that CL tumors are the most undifferentiated ones at the molecular level along the normal mammary epithelial differentiation hierarchy (differentiation score close the stem cell profile) and are the most enriched in stem cell features, followed by basal tumors. CL and basal tumors are also distinguished by the expression of several pathway activation signatures; for examples, basal cancers displayed higher activity of MYC and PI3K pathways as already reported [60, 61], whereas CL tumors showed higher activity of EGFR, SRC and TGFβ pathways as reported by others [7] with therapeutic possibilities.

Regarding prognosis, the DFS, available for 343 patients with CL BC, was poor with 67% 5-year DFS, close to that reported by Prat and colleagues in their 58-patients series [9]. Compared with the other subtypes, the 5-year DFS was inferior to that observed in the two good-prognosis subtypes (luminal A and normal-like), similar to that observed in the luminal B subtype, and tended to be better than that observed in the two other poor-prognosis subtypes (basal and ERBB2-enriched), even if the difference (7%) with basal tumors was not significant. The earlier timing of relapse compared with the luminal A subtype, similarly to that observed in the basal subtype, agreed with the proliferative nature of CL tumors. It is now recognized that the prognostic features are somewhat different between the different subtypes [62, 63]. This has never been explored to date in CL BCs. Most of the clinicopathological prognostic variables tested in CL samples were significant (pT, grade, pN, *ESR1 and PGR* expression), as observed in the whole series and the luminal A subtype. Unexpectedly, in term of prognostic features, the subtype most different from CL was the basal subtype, and the most similar was the luminal A subtype, the less proliferative one. We also analyzed the prognostic value of 8 published signatures, 4 related to cell proliferation, known to be strongly prognostic in ER-positive samples, and 4 to immunity, known to be strongly prognostic in ER-negative samples. Similarly to the results observed with the clinicopathological variables, proliferation-related classifiers useful to predict luminal A tumors prognosis had (or tended to have) a prognostic value in CL samples, whereas immunity-related GES, described to be relevant in basal and HER2-enriched BCs, did not.

The profile of chemosensitivity of CL tumors was assessed in 228 cases. The pCR rate after neoadjuvant chemotherapy was close (32%) to that of basal (33%) and ERBB2-enriched tumors (37%), and significantly higher than the rates observed for luminal A (7%), luminal B (18%), and normal-like tumors (14%). Similar observations have been reported by Prat and colleagues [9] in a smaller series of 133 samples including 18 CL, with higher pCR rates in the CL (39%), basal (79%) and ERBB2-enriched (39%) cases. As discussed above with the prognostic features, the clinicopathological features predictive for pCR in the CL subtype (grade and *ESR1* status) were predictive in the whole series of samples and the luminal A subtype, but were not predictive in the ER-negative subtypes (basal and ERBB2-enriched). The predictive value of 4 previously published predictive GES was also different between CL and the other subtypes.

## Conclusions

In conclusion, we revealed many differences between CL and the other molecular subtype of breast cancers, notably the basal subtype. Differences are present at all tested levels, including the molecular and clinicopathological characteristics, the clinical outcome and the prognostic features. The strength of our study lies in both its comprehensiveness and the number of samples analyzed. Limitations are those of any retrospective multicenter study, including potential selection bias and the unavailability of certain clinicopathological variables. Our results suggest that CL tumors represent a different subtype. They also reveal some unexpected findings that warrant further study to better understand this yet mysterious subtype. The most important ones concern the relatively high numbers of ER-positive tumors and non-TN tumors within the CL subtype: 36% and 48% respectively *versus* only 14% and 24% in the basal subtype and 95% and 99% in the luminal A subtype. This difference in a major feature of breast cancer (ER status) suggests that the so-defined CL subtype is much more heterogeneous than basal and luminal A subtypes. Such a mixture of ER-negative and ER-positive samples likely explains the intermediate/mixed pattern of CL subtype between the basal and the luminal A subtypes in terms of clinicopathological and molecular characteristics, survival, prognostic features, and response to chemotherapy.

## Electronic supplementary material

Additional file 1: Table S1: Description of the 32 breast cancer data sets. (XLSX 19 KB)

Additional file 2: **Sweave report of gene expression data and associated statistics.** (PDF 279 KB)

Additional file 3: Table S2: Genomic patterns of the molecular subtypes using the Hicks’ classification [56]. (XLSX 10 KB)

Additional file 4: Figure S1: Comparison of probabilities of pathway activation across molecular subtypes. Box plots of probabilities of activation of 12 pathways from [44]. P-values (*t*-test) of comparisons between CL and each of the other subtypes are shown as follows: *, ≤5%; **, ≤1%; ***, ≤0.1%. (PDF 11 KB)

Additional file 5: Table S3: Odd ratios and *p*-values of comparison of gene expression signatures across molecular subtypes. (XLSX 11 KB)

Additional file 6: Figure S2: Comparison of mRNA expression levels of genes associated with EMT across molecular subtypes. Expression values are log2-scaled. P-values (*t*-test) of comparisons between CL and each of the other subtypes are shown as follows: *, ≤5%; **, ≤1%; ***, ≤0.1%. (PDF 11 KB)

Additional file 7: Table S4: Univariate Cox regression analysis for DFS in all subtypes. (XLSX 18 KB)

Additional file 8: Table S5: Univariate analysis for pathological complete response according to clinicopathological and molecular features in all subtypes. (XLSX 22 KB)

## Abbreviations

CL: Claudin-low
BC: Breast cancer
EMT: Epithelial to mesenchymal transition
ER: Estrogen receptor
PR: Progesterone receptor
DFS: Disease-free survival
pCR: Pathological complete response
GES: Gene expression signature
TN: Triple-negative
RMA: Robust multichip average
IHC: Immunohistochemistry
IPC: Institut Paoli-Calmettes
GGI: Genomic grade index
RS: Recurrence score
ROR: Risk of relapse
DLDA-30: Diagonal Linear Discriminant Analysis–30
CGH: Comparative genomic hybridization
CNA: Copy number alterations.

## References

[1] Viale G (2012). The current state of breast cancer classification. Ann Oncol.

[2] Perou CM, Sørlie T, Eisen MB, van de Rijn M, Jeffrey SS, Rees CA, Pollack JR, Ross DT, Johnsen H, Akslen LA, Fluge O, Pergamenschikov A, Williams C, Zhu SX, Lønning PE, Børresen-Dale AL, Brown PO, Botstein D (2000). Molecular portraits of human breast tumours. Nature.

[3] Veer LJ V’t, Dai H, van de Vijver MJ, He YD, Hart AAM, Mao M, Peterse HL, van der Kooy K, Marton MJ, Witteveen AT, Schreiber GJ, Kerkhoven RM, Roberts C, Linsley PS, Bernards R, Friend SH (2002). Gene expression profiling predicts clinical outcome of breast cancer. Nature.

[4] Sørlie T, Perou CM, Tibshirani R, Aas T, Geisler S, Johnsen H, Hastie T, Eisen MB, van de Rijn M, Jeffrey SS, Thorsen T, Quist H, Matese JC, Brown PO, Botstein D, Lønning PE, Børresen-Dale AL (2001). Gene expression patterns of breast carcinomas distinguish tumor subclasses with clinical implications. Proc Natl Acad Sci U S A.

[5] Hu Z, Fan C, Oh DS, Marron JS, He X, Qaqish BF, Livasy C, Carey LA, Reynolds E, Dressler L, Nobel A, Parker J, Ewend MG, Sawyer LR, Wu J, Liu Y, Nanda R, Tretiakova M, Ruiz Orrico A, Dreher D, Palazzo JP, Perreard L, Nelson E, Mone M, Hansen H, Mullins M, Quackenbush JF, Ellis MJ, Olopade OI, Bernard PS, Perou CM (2006). The molecular portraits of breast tumors are conserved across microarray platforms. BMC Genomics.

[6] Farmer P, Bonnefoi H, Becette V, Tubiana-Hulin M, Fumoleau P, Larsimont D, Macgrogan G, Bergh J, Cameron D, Goldstein D, Duss S, Nicoulaz A-L, Brisken C, Fiche M, Delorenzi M, Iggo R (2005). Identification of molecular apocrine breast tumours by microarray analysis. Oncogene.

[7] Lehmann BD, Bauer JA, Chen X, Sanders ME, Chakravarthy AB, Shyr Y, Pietenpol JA (2011). Identification of human triple-negative breast cancer subtypes and preclinical models for selection of targeted therapies. J Clin Invest.

[8] Herschkowitz JI, Simin K, Weigman VJ, Mikaelian I, Usary J, Hu Z, Rasmussen KE, Jones LP, Assefnia S, Chandrasekharan S, Backlund MG, Yin Y, Khramtsov AI, Bastein R, Quackenbush J, Glazer RI, Brown PH, Green JE, Kopelovich L, Furth PA, Palazzo JP, Olopade OI, Bernard PS, Churchill GA, Van Dyke T, Perou CM (2007). Identification of conserved gene expression features between murine mammary carcinoma models and human breast tumors. Genome Biol.

[9] Prat A, Parker JS, Karginova O, Fan C, Livasy C, Herschkowitz JI, He X, Perou CM (2010). Phenotypic and molecular characterization of the claudin-low intrinsic subtype of breast cancer. Breast Cancer Res.

[10] Creighton CJ, Li X, Landis M, Dixon JM, Neumeister VM, Sjolund A, Rimm DL, Wong H, Rodriguez A, Herschkowitz JI, Fan C, Zhang X, He X, Pavlick A, Gutierrez MC, Renshaw L, Larionov AA, Faratian D, Hilsenbeck SG, Perou CM, Lewis MT, Rosen JM, Chang JC (2009). Residual breast cancers after conventional therapy display mesenchymal as well as tumor-initiating features. Proc Natl Acad Sci U S A.

[11] Van de Vijver MJ, He YD, van’t Veer LJ, Dai H, Hart AAM, Voskuil DW, Schreiber GJ, Peterse JL, Roberts C, Marton MJ, Parrish M, Atsma D, Witteveen A, Glas A, Delahaye L, van der Velde T, Bartelink H, Rodenhuis S, Rutgers ET, Friend SH, Bernards R (2002). A gene-expression signature as a predictor of survival in breast cancer. N Engl J Med.

[12] Schmidt M, Böhm D, von Törne C, Steiner E, Puhl A, Pilch H, Lehr H-A, Hengstler JG, Kölbl H, Gehrmann M (2008). The humoral immune system has a key prognostic impact in node-negative breast cancer. Cancer Res.

[13] Wang Y, Klijn JGM, Zhang Y, Sieuwerts AM, Look MP, Yang F, Talantov D, Timmermans M, Meijer-van Gelder ME, Yu J, Jatkoe T, Berns EMJJ, Atkins D, Foekens JA (2005). Gene-expression profiles to predict distant metastasis of lymph-node-negative primary breast cancer. Lancet.

[14] Sotiriou C, Wirapati P, Loi S, Harris A, Fox S, Smeds J, Nordgren H, Farmer P, Praz V, Haibe-Kains B, Desmedt C, Larsimont D, Cardoso F, Peterse H, Nuyten D, Buyse M, Van de Vijver MJ, Bergh J, Piccart M, Delorenzi M (2006). Gene expression profiling in breast cancer: understanding the molecular basis of histologic grade to improve prognosis. J Natl Cancer Inst.

[15] Ivshina AV, George J, Senko O, Mow B, Putti TC, Smeds J, Lindahl T, Pawitan Y, Hall P, Nordgren H, Wong JEL, Liu ET, Bergh J, Kuznetsov VA, Miller LD (2006). Genetic reclassification of histologic grade delineates new clinical subtypes of breast cancer. Cancer Res.

[16] Desmedt C, Piette F, Loi S, Wang Y, Lallemand F, Haibe-Kains B, Viale G, Delorenzi M, Zhang Y, d’ Assignies MS, Bergh J, Lidereau R, Ellis P, Harris AL, Klijn JGM, Foekens JA, Cardoso F, Piccart MJ, Buyse M, Sotiriou C (2007). Strong time dependence of the 76-gene prognostic signature for node-negative breast cancer patients in the TRANSBIG multicenter independent validation series. Clin Cancer Res.

[17] Miller LD, Smeds J, George J, Vega VB, Vergara L, Ploner A, Pawitan Y, Hall P, Klaar S, Liu ET, Bergh J (2005). An expression signature for p53 status in human breast cancer predicts mutation status, transcriptional effects, and patient survival. Proc Natl Acad Sci U S A.

[18] Hess KR, Anderson K, Symmans WF, Valero V, Ibrahim N, Mejia JA, Booser D, Theriault RL, Buzdar AU, Dempsey PJ, Rouzier R, Sneige N, Ross JS, Vidaurre T, Gómez HL, Hortobagyi GN, Pusztai L (2006). Pharmacogenomic predictor of sensitivity to preoperative chemotherapy with paclitaxel and fluorouracil, doxorubicin, and cyclophosphamide in breast cancer. J Clin Oncol.

[19] Bonnefoi H, Potti A, Delorenzi M, Mauriac L, Campone M, Tubiana-Hulin M, Petit T, Rouanet P, Jassem J, Blot E, Becette V, Farmer P, André S, Acharya CR, Mukherjee S, Cameron D, Bergh J, Nevins JR, Iggo RD (2007). Validation of gene signatures that predict the response of breast cancer to neoadjuvant chemotherapy: a substudy of the EORTC 10994/BIG 00–01 clinical trial. Lancet Oncol.

[20] Guedj M, Marisa L, de Reynies A, Orsetti B, Schiappa R, Bibeau F, MacGrogan G, Lerebours F, Finetti P, Longy M, Bertheau P, Bertrand F, Bonnet F, Martin AL, Feugeas JP, Bièche I, Lehmann-Che J, Lidereau R, Birnbaum D, Bertucci F, de Thé H, Theillet C (2012). A refined molecular taxonomy of breast cancer. Oncogene.

[21] Desmedt C, Di Leo A, de Azambuja E, Larsimont D, Haibe-Kains B, Selleslags J, Delaloge S, Duhem C, Kains J-P, Carly B, Maerevoet M, Vindevoghel A, Rouas G, Lallemand F, Durbecq V, Cardoso F, Salgado R, Rovere R, Bontempi G, Michiels S, Buyse M, Nogaret J-M, Qi Y, Symmans F, Pusztai L, D’Hondt V, Piccart-Gebhart M, Sotiriou C (2011). Multifactorial approach to predicting resistance to anthracyclines. J Clin Oncol.

[22] **Expression Project for Oncology** [http://www.ncbi.nlm.nih.gov/geo/query/acc.cgi?acc=GSE2109]

[23] Korde LA, Lusa L, McShane L, Lebowitz PF, Lukes L, Camphausen K, Parker JS, Swain SM, Hunter K, Zujewski JA (2010). Gene expression pathway analysis to predict response to neoadjuvant docetaxel and capecitabine for breast cancer. Breast Cancer Res Treat.

[24] Minn AJ, Gupta GP, Siegel PM, Bos PD, Shu W, Giri DD, Viale A, Olshen AB, Gerald WL, Massagué J (2005). Genes that mediate breast cancer metastasis to lung. Nature.

[25] Miller WR, Larionov AA, Renshaw L, Anderson TJ, White S, Murray J, Murray E, Hampton G, Walker JR, Ho S, Krause A, Evans DB, Dixon JM (2007). Changes in breast cancer transcriptional profiles after treatment with the aromatase inhibitor, letrozole. Pharmacogenet Genomics.

[26] Klein A, Wessel R, Graessmann M, Jürgens M, Petersen I, Schmutzler R, Niederacher D, Arnold N, Meindl A, Scherneck S, Seitz S, Graessmann A (2007). Comparison of gene expression data from human and mouse breast cancers: identification of a conserved breast tumor gene set. Int J Cancer.

[27] Hoeflich KP, O’Brien C, Boyd Z, Cavet G, Guerrero S, Jung K, Januario T, Savage H, Punnoose E, Truong T, Zhou W, Berry L, Murray L, Amler L, Belvin M, Friedman LS, Lackner MR (2009). In vivo antitumor activity of MEK and phosphatidylinositol 3-kinase inhibitors in basal-like breast cancer models. Clin Cancer Res.

[28] Marty B, Maire V, Gravier E, Rigaill G, Vincent-Salomon A, Kappler M, Lebigot I, Djelti F, Tourdès A, Gestraud P, Hupé P, Barillot E, Cruzalegui F, Tucker GC, Stern M-H, Thiery J-P, Hickman JA, Dubois T (2008). Frequent PTEN genomic alterations and activated phosphatidylinositol 3-kinase pathway in basal-like breast cancer cells. Breast Cancer Res.

[29] Chin K, DeVries S, Fridlyand J, Spellman PT, Roydasgupta R, Kuo W-L, Lapuk A, Neve RM, Qian Z, Ryder T, Chen F, Feiler H, Tokuyasu T, Kingsley C, Dairkee S, Meng Z, Chew K, Pinkel D, Jain A, Ljung BM, Esserman L, Albertson DG, Waldman FM, Gray JW (2006). Genomic and transcriptional aberrations linked to breast cancer pathophysiologies. Cancer Cell.

[30] Yu K, Ganesan K, Tan LK, Laban M, Wu J, Zhao XD, Li H, Leung CHW, Zhu Y, Wei CL, Hooi SC, Miller L, Tan P (2008). A precisely regulated gene expression cassette potently modulates metastasis and survival in multiple solid cancers. PLoS Genet.

[31] Bos PD, Zhang XH-F, Nadal C, Shu W, Gomis RR, Nguyen DX, Minn AJ, van de Vijver MJ, Gerald WL, Foekens JA, Massagué J (2009). Genes that mediate breast cancer metastasis to the brain. Nature.

[32] Zhang Y, Sieuwerts AM, McGreevy M, Casey G, Cufer T, Paradiso A, Harbeck N, Span PN, Hicks DG, Crowe J, Tubbs RR, Budd GT, Lyons J, Sweep FCGJ, Schmitt M, Schittulli F, Golouh R, Talantov D, Wang Y, Foekens JA (2009). The 76-gene signature defines high-risk patients that benefit from adjuvant tamoxifen therapy. Breast Cancer Res Treat.

[33] Barry WT, Kernagis DN, Dressman HK, Griffis RJ, Hunter JD, Olson JA, Marks JR, Ginsburg GS, Marcom PK, Nevins JR, Geradts J, Datto MB (2010). Intratumor heterogeneity and precision of microarray-based predictors of breast cancer biology and clinical outcome. J Clin Oncol.

[34] Silver DP, Richardson AL, Eklund AC, Wang ZC, Szallasi Z, Li Q, Juul N, Leong C-O, Calogrias D, Buraimoh A, Fatima A, Gelman RS, Ryan PD, Tung NM, De Nicolo A, Ganesan S, Miron A, Colin C, Sgroi DC, Ellisen LW, Winer EP, Garber JE (2010). Efficacy of neoadjuvant Cisplatin in triple-negative breast cancer. J Clin Oncol.

[35] Chen D-T, Nasir A, Culhane A, Venkataramu C, Fulp W, Rubio R, Wang T, Agrawal D, McCarthy SM, Gruidl M, Bloom G, Anderson T, White J, Quackenbush J, Yeatman T (2010). Proliferative genes dominate malignancy-risk gene signature in histologically-normal breast tissue. Breast Cancer Res Treat.

[36] Popovici V, Chen W, Gallas BG, Hatzis C, Shi W, Samuelson FW, Nikolsky Y, Tsyganova M, Ishkin A, Nikolskaya T, Hess KR, Valero V, Booser D, Delorenzi M, Hortobagyi GN, Shi L, Symmans WF, Pusztai L (2010). Effect of training-sample size and classification difficulty on the accuracy of genomic predictors. Breast Cancer Res.

[37] Iwamoto T, Bianchini G, Booser D, Qi Y, Coutant C, Shiang CY-H, Santarpia L, Matsuoka J, Hortobagyi GN, Symmans WF, Holmes FA, O’Shaughnessy J, Hellerstedt B, Pippen J, Andre F, Simon R, Pusztai L (2011). Gene pathways associated with prognosis and chemotherapy sensitivity in molecular subtypes of breast cancer. J Natl Cancer Inst.

[38] Tabchy A, Valero V, Vidaurre T, Lluch A, Gomez H, Martin M, Qi Y, Barajas-Figueroa LJ, Souchon E, Coutant C, Doimi FD, Ibrahim NK, Gong Y, Hortobagyi GN, Hess KR, Symmans WF, Pusztai L (2010). Evaluation of a 30-gene paclitaxel, fluorouracil, doxorubicin, and cyclophosphamide chemotherapy response predictor in a multicenter randomized trial in breast cancer. Clin Cancer Res.

[39] Hatzis C, Pusztai L, Valero V, Booser DJ, Esserman L, Lluch A, Vidaurre T, Holmes F, Souchon E, Wang H, Martin M, Cotrina J, Gomez H, Hubbard R, Chacón JI, Ferrer-Lozano J, Dyer R, Buxton M, Gong Y, Wu Y, Ibrahim N, Andreopoulou E, Ueno NT, Hunt K, Yang W, Nazario A, DeMichele A, O’Shaughnessy J, Hortobagyi GN, Symmans WF (2011). A genomic predictor of response and survival following taxane-anthracycline chemotherapy for invasive breast cancer. JAMA.

[40] Irizarry RA, Hobbs B, Collin F, Beazer-Barclay YD, Antonellis KJ, Scherf U, Speed TP (2003). Exploration, normalization, and summaries of high density oligonucleotide array probe level data. Biostat Oxf Engl.

[41] Parker JS, Mullins M, Cheang MCU, Leung S, Voduc D, Vickery T, Davies S, Fauron C, He X, Hu Z, Quackenbush JF, Stijleman IJ, Palazzo J, Marron JS, Nobel AB, Mardis E, Nielsen TO, Ellis MJ, Perou CM, Bernard PS (2009). Supervised risk predictor of breast cancer based on intrinsic subtypes. J Clin Oncol.

[42] Palmer C, Diehn M, Alizadeh AA, Brown PO (2006). Cell-type specific gene expression profiles of leukocytes in human peripheral blood. BMC Genomics.

[43] Taube JH, Herschkowitz JI, Komurov K, Zhou AY, Gupta S, Yang J, Hartwell K, Onder TT, Gupta PB, Evans KW, Hollier BG, Ram PT, Lander ES, Rosen JM, Weinberg RA, Mani SA (2010). Core epithelial-to-mesenchymal transition interactome gene-expression signature is associated with claudin-low and metaplastic breast cancer subtypes. Proc Natl Acad Sci U S A.

[44] Gatza ML, Lucas JE, Barry WT, Kim JW, Wang Q, Crawford MD, Datto MB, Kelley M, Mathey-Prevot B, Potti A, Nevins JR (2010). A pathway-based classification of human breast cancer. Proc Natl Acad Sci U S A.

[45] Lim E, Vaillant F, Wu D, Forrest NC, Pal B, Hart AH, Asselin-Labat M-L, Gyorki DE, Ward T, Partanen A, Feleppa F, Huschtscha LI, Thorne HJ, Fox SB, Yan M, French JD, Brown MA, Smyth GK, Visvader JE, Lindeman GJ (2009). Aberrant luminal progenitors as the candidate target population for basal tumor development in BRCA1 mutation carriers. Nat Med.

[46] Paik S, Shak S, Tang G, Kim C, Baker J, Cronin M, Baehner FL, Walker MG, Watson D, Park T, Hiller W, Fisher ER, Wickerham DL, Bryant J, Wolmark N (2004). A multigene assay to predict recurrence of tamoxifen-treated, node-negative breast cancer. N Engl J Med.

[47] Bianchini G, Qi Y, Alvarez RH, Iwamoto T, Coutant C, Ibrahim NK, Valero V, Cristofanilli M, Green MC, Radvanyi L, Hatzis C, Hortobagyi GN, Andre F, Gianni L, Symmans WF, Pusztai L (2010). Molecular anatomy of breast cancer stroma and its prognostic value in estrogen receptor-positive and -negative cancers. J Clin Oncol.

[48] Sabatier R, Finetti P, Mamessier E, Raynaud S, Cervera N, Lambaudie E, Jacquemier J, Viens P, Birnbaum D, Bertucci F (2011). Kinome expression profiling and prognosis of basal breast cancers. Mol Cancer.

[49] Rody A, Holtrich U, Pusztai L, Liedtke C, Gaetje R, Ruckhaeberle E, Solbach C, Hanker L, Ahr A, Metzler D, Engels K, Karn T, Kaufmann M (2009). T-cell metagene predicts a favorable prognosis in estrogen receptor-negative and HER2-positive breast cancers. Breast Cancer Res.

[50] Teschendorff AE, Miremadi A, Pinder SE, Ellis IO, Caldas C (2007). An immune response gene expression module identifies a good prognosis subtype in estrogen receptor negative breast cancer. Genome Biol.

[51] Farmer P, Bonnefoi H, Anderle P, Cameron D, Wirapati P, Wirapati P, Becette V, André S, Piccart M, Campone M, Brain E, Macgrogan G, Petit T, Jassem J, Bibeau F, Blot E, Bogaerts J, Aguet M, Bergh J, Iggo R, Delorenzi M (2009). A stroma-related gene signature predicts resistance to neoadjuvant chemotherapy in breast cancer. Nat Med.

[52] Ertel A, Dean JL, Rui H, Liu C, Witkiewicz AK, Knudsen KE, Knudsen ES (2010). RB-pathway disruption in breast cancer: differential association with disease subtypes, disease-specific prognosis and therapeutic response. Cell Cycle Georget Tex.

[53] Adélaïde J, Finetti P, Bekhouche I, Repellini L, Geneix J, Sircoulomb F, Charafe-Jauffret E, Cervera N, Desplans J, Parzy D, Schoenmakers E, Viens P, Jacquemier J, Birnbaum D, Bertucci F, Chaffanet M (2007). Integrated profiling of basal and luminal breast cancers. Cancer Res.

[54] Beroukhim R, Getz G, Nghiemphu L, Barretina J, Hsueh T, Linhart D, Vivanco I, Lee JC, Huang JH, Alexander S, Du J, Kau T, Thomas RK, Shah K, Soto H, Perner S, Prensner J, Debiasi RM, Demichelis F, Hatton C, Rubin MA, Garraway LA, Nelson SF, Liau L, Mischel PS, Cloughesy TF, Meyerson M, Golub TA, Lander ES, Mellinghoff IK, Sellers WR (2007). Assessing the significance of chromosomal aberrations in cancer: methodology and application to glioma. Proc Natl Acad Sci U S A.

[55] Herschkowitz JI, Zhao W, Zhang M, Usary J, Murrow G, Edwards D, Knezevic J, Greene SB, Darr D, Troester MA, Hilsenbeck SG, Medina D, Perou CM, Rosen JM (2011). Comparative oncogenomics identifies breast tumors enriched in functional tumor-initiating cells. Proc Natl Acad Sci.

[56] Hicks J, Krasnitz A, Lakshmi B, Navin NE, Riggs M, Leibu E, Esposito D, Alexander J, Troge J, Grubor V, Yoon S, Wigler M, Ye K, Børresen-Dale A-L, Naume B, Schlicting E, Norton L, Hägerström T, Skoog L, Auer G, Månér S, Lundin P, Zetterberg A (2006). Novel patterns of genome rearrangement and their association with survival in breast cancer. Genome Res.

[57] McShane LM, Altman DG, Sauerbrei W, Taube SE, Gion M, Clark GM (2005). Reporting recommendations for tumor marker prognostic studies (REMARK). J Natl Cancer Inst.

[58] Gong Y, Yan K, Lin F, Anderson K, Sotiriou C, Andre F, Holmes FA, Valero V, Booser D, Pippen JE, Vukelja S, Gomez H, Mejia J, Barajas LJ, Hess KR, Sneige N, Hortobagyi GN, Pusztai L, Symmans WF (2007). Determination of oestrogen-receptor status and ERBB2 status of breast carcinoma: a gene-expression profiling study. Lancet Oncol.

[59] Di Fiore PP, Pierce JH, Kraus MH, Segatto O, King CR, Aaronson SA (1987). erbB-2 is a potent oncogene when overexpressed in NIH/3 T3 cells. Science.

[60] The Cancer Genome Atlas Network (2012). Comprehensive molecular portraits of human breast tumours. Nature.

[61] Chandriani S, Frengen E, Cowling VH, Pendergrass SA, Perou CM, Whitfield ML, Cole MD (2009). A core MYC gene expression signature is prominent in basal-like breast cancer but only partially overlaps the core serum response. PLoS One.

[62] Bertucci F, Birnbaum D (2012). [Genomics and clinical research for breast cancer]. Médecine Sci MS.

[63] Bertucci F, Finetti P, Birnbaum D (2012). Basal breast cancer: a complex and deadly molecular subtype. Curr Mol Med.

