# Supplementary material for: Claudin-low breast cancers: clinical, pathological, molecular and prognostic characterization
Source: Mol Cancer. 2014 Oct 2;13:228. doi: 10.1186/1476-4598-13-228 (PMC4197217; doi:10.1186/1476-4598-13-228)
Supplement: Supplementary file 2 — Additional file 2: Sweave report of gene expression data and associated statistics. (PDF 279 KB) [file 12943_2014_1429_MOESM2_ESM.pdf]

# Claudin-low breast cancers: clinical, pathological, molecular and prognostic characterization

...

## Sweave report

...

Renaud Sabatier, Pascal Finetti, Arnaud Guille, José Adelaïde, Max Chaffanet, Patrice Viens, Daniel Birnbaum, François Bertucci

# 1 Preparation of public data

## 1.1 Loading description file sets with their location:

```
> library(xtable)
> library(affy)
> library(e1071)
> Path <- "G:/D_Old/DATA/CLDN/20140404_Papier_RS/20140807_retourRev/docs_Prep_SweaveR/Prep_Obj_Sweav
> Set <- read.delim(paste(Path, "Description_32sets.txt", sep="/"))
> print(xtable(Set[,c(3,5,7)]), size="footnotesize",include.rownames = FALSE)
```

| File_Name | DataSet_ID | Platform |
|---|---|---|
| 2006.Ivshina06.Upp.Sing.Stock.448BC.GSE4922_1456.RMA.RData | GSE4922_1456 | hgu133ab |
| 2006.Sotiriou.189BC.GSE2990.RMA.RData | GSE2990 | hgu133a |
| 2008.Schmidt.200BC.GSE11121.RMA.RData | GSE11121 | hgu133a |
| 2007.Desmedt.198BC.GSE7390.RMA.RData | GSE7390 | hgu133a |
| 2002.van.t.Veer.117BC.Proc.RData | vVeer | vVeerchip |
| 2002.van.de.Vijver.295BC.Proc.RData | vVijver | vVijverchip |
| 2005.Wang.286BC.GSE2034.RMA.RData | GSE2034 | hgu133a |
| 2006.Hess.133BC.MDA133.RMA.RData | MDA133 | hgu133a |
| IPC372_RMA_S4.RData | IPC_BCSet | hgu133plus2 |
| 2007.Bonnefoi.161BC.GSE6861.RMA.RData | GSE6861 | hgu133x3p |
| 2011.Hatzis.GSE25066.508BC.RMA.RData | GSE25066 | hgu133a |
| 2011.Guedj.EMTAB365.537BC.RMA.RData | ETABM365 | hgu133plus2 |
| 2011.Desmedt.GSE16446.120BC.RMA.RData | GSE16446 | hgu133plus2 |
| 2005.ExpO.GSE2109.348BC.RMA.RData | GSE2109 | hgu133plus2 |
| 2005.Farmer.GSE1561.49BC.RMA.RData | GSE1561 | hgu133a |
| 2005.Minn.GSE2603.99BC.RMA.RData | GSE2603 | hgu133a |
| 2007.Miller.GSE5462.58x2BC.RMA.RData | GSE5462 | hgu133a |
| 2007.Seitz.GSE6596.24BCs2NL.RMA.RData | GSE6596 | hgu133a |
| 2008.Hoeflich.GSE12763.30BC.RMA.RData | GSE12763 | hgu133plus2 |
| 2008.Marty.GSE13787.23BC.RMA.RData | GSE13787 | hgu133plus2 |
| 2008.Merritt.ETABM158.130BC.RMA.RData | ETABM158 | u133aaofav2 |
| 2008.Yu.GSE5364.183BC13NL_MAS5.RData | GSE5364 | hgu133a |
| 2009.Bos.GSE12276.204BC.RMA.RData | GSE12276 | hgu133plus2 |
| 2009.Zhang.GSE12093.136BC.RMA.RData | GSE12093 | hgu133a |
| 2010.Barry.GSE23593.50BC.18x2ou3.RMA.RData | GSE23593 | hgu133plus2 |
| 2010.Korde.GSE18728.30BC.61echts.RMA.RData | GSE18728 | hgu133plus2 |
| 2010.Prat.GSE18229.eSet.337BC.RMA.RData | GSE18229 | GPLPrat |
| 2010.Silver.GSE18864.84BC9repl.RMA.RData | GSE18864 | hgu133plus2 |
| 2011.Chen.GSE10780.42BC143pNl.RMA.RData | GSE10780 | hgu133plus2 |
| 2012.Popovoci.GSE20194.278BC.RMA.RData | GSE20194 | hgu133a |
| 2010.Iwamoto.PoolGSE22093_23988.RMA.RData | GSE22093_GSE23988 | hgu133a |
| 2010.Tabchy.GSE20271.RMA.RData | GSE20271 | hgu133a |

Public sets[1]

[1]Each of .RData objects listed were previously constructed from expression data and phenodata available. Phenodata were cleaned and homogenized across data sets. For Agilent-based data sets, we applied quantile normalization (*limma* package) to processed data. For Affymetrix-based data sets, we used Robust Multichip Average (RMA, *affy* package) on the raw .CEL files. Updated genechips annotation were integrated in their respective .RData object.

## 1.2 Merging phenodata from .RData objects

```
> PublicSet <- list(HC=c()) ; require(affy)
> for (i in seq_along(Set$Nom_ObjHC)){
+   load(paste(Path, "RData_ObjSets", Set$File_Name[i], sep="/"))
+   PublicSet$HC <- rbind(PublicSet$HC, pData(Eset_Obj)) ; rm(Eset_Obj)
+ }
> print(xtable(data.frame(table(PublicSet$HC$SetName)),
+              caption="Merged phenodata effectif"),
+       size="footnotesize",
+       include.rownames = FALSE)
```

| Var1 | Freq |
|---|---|
| 2006.Ivshina.448BC.GSE4922.1456 | 448 |
| 2006.Sotiriou.189BC.GSE2990 | 189 |
| 2008.Schmidt.200BC.GSE11121 | 200 |
| 2007.Desmedt.198BC.GSE7390 | 198 |
| 2002.van.t.Veer.117BC | 117 |
| 2002.van.de.Vijver.295BC | 295 |
| 2005.Wang.286BC.GSE2034 | 286 |
| 2006.Hess.133BC.MDA133 | 133 |
| IPC372_RMA_S4.RData | 372 |
| 2007.Bonnefoi.161BC.GSE6861 | 161 |
| 2011.Hatzis.GSE25066.508BC | 508 |
| 2011.Guedj.EMTAB365.537BC | 537 |
| 2011.Desmedt.GSE16446.120BC | 120 |
| 2005.ExpO.GSE2109.348BC | 348 |
| 2005.Farmer.GSE1561.49BC | 49 |
| 2005.Minn.GSE2603.99BC | 99 |
| 2007.Miller.GSE5462.58x2BC | 116 |
| 2007.Seitz.GSE6596.24BCs2NL | 26 |
| 2008.Hoeflich.GSE12763.30BC | 30 |
| 2008.Marty.GSE13787.23BC | 23 |
| 2008.Merritt.ETABM158.130BC | 130 |
| 2008.Yu.GSE5364.183BC13NL | 196 |
| 2009.Bos.GSE12276.204BC | 204 |
| 2009.Zhang.GSE12093.136BC | 136 |
| 2010.Barry.GSE23593.50BC.18x23 | 50 |
| 2010.Korde.GSE18728.30BC.61e | 61 |
| 2010.Prat.GSE18229.337BC | 337 |
| 2010.Silver.GSE18864.84BC9repl | 84 |
| 2011.Chen.GSE10780.42BC143pNl | 185 |
| 2012.Popovoci.GSE20194.278BC | 278 |
| 2010.Iwamoto.GSE22093.23988.164 | 164 |
| 2010.Tabchy.GSE20271.178BC | 178 |

Merged phenodata effectif

```
> nrow(PublicSet$HC)
```

[1] 6258

## 1.3 Selection of primary breast cancer samples without duplicate mesurement

```
> sel_Primaire <- which(PublicSet$HC$Type %in% "PrimaryBC"
+                       & PublicSet$HC$Duplicate %in% "unique")
> length(sel_Primaire)
```

```
[1] 5447
```

# 2 Export markers & classification

## 2.1 Export markers (ESR1, PGR, ERBB2) & GMM groups:

Define genes by their Entrez Gene ID for matching

```
> ref_EntrezGeneID <- list(ESR1="2099", PGR="5241", ERBB2="2064")
```

Probes selection based on signal levels and variance

```
> source("G:\\Ori Tools\\Prog\\Rwork\\Script\\20120828_ExportGene_S4Obj.r")
>   # batch function
> VAR_GeneCtrl <- Batch_ExportGene_PF(ref_EntrezGeneID, SET=Set)
```

Gaussian Mixture Models (GMM) segmentation function based on the mixtools R package

```
> source("G:/Ori Tools/Prog/Rwork/Script/20130118_GMMnK_EMsegmentation.r")
> GMM_Sets <- list()
> require(limma)
> for (i in seq_along(VAR_GeneCtrl)){
+   cat(paste(names(VAR_GeneCtrl)[i], ":"))
+   GMM_Sets[[i]] <- Seg_EM_PF(VAR_GeneCtrl[[i]]$Expres, Nom=names(VAR_GeneCtrl)[i])
+   cat("done.\n\n")
+ }
```

Plot GMM from 3 random sets:

```
> require(mixtools)
> par(mfrow=c(3,3))
>  for (i in sample(seq(32),3)){
+   for(j in seq(3)){
+     plot(GMM_Sets[[i]][[j]]$mixmdl, which=2, las=1,
+          main2=names(GMM_Sets[[i]])[j],
+          col2=c(3,2), xlab2=names(GMM_Sets)[i])
+     abline(v=GMM_Sets[[i]][[j]]$Cut_off, col="orange", lty=2)
+   }
+ }
```

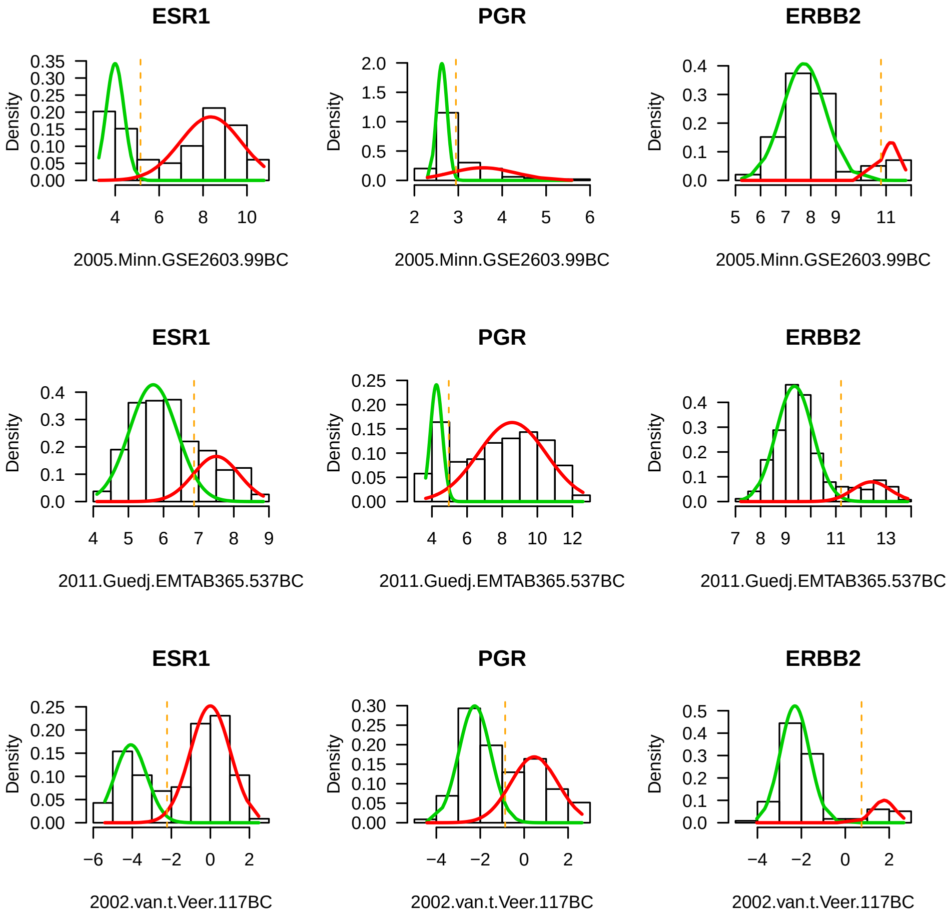


GMM segmentation & merge *ESR1*, *PGR* & *ERBB2* genes

```
> VAR_Gene <- c()
> for (i in seq_along(GMM_Sets)){
+      VAR_Gene <- rbind(VAR_Gene, cbind(
+          ESR1=ifelse(VAR_GeneCtrl[[i]]$Expres$ESR1 >
+                        GMM_Sets[[i]]$ESR1$Cut_off, 1,
+                      ifelse(VAR_GeneCtrl[[i]]$Expres$ESR1 <=
+                                 GMM_Sets[[i]]$ESR1$Cut_off, 0, NA )),
+          PGR=ifelse(VAR_GeneCtrl[[i]]$Expres$PGR >
+                       GMM_Sets[[i]]$PGR$Cut_off, 1,
+                     ifelse(VAR_GeneCtrl[[i]]$Expres$PGR <=
+                                 GMM_Sets[[i]]$PGR$Cut_off, 0, NA )),
+          ERBB2=ifelse(VAR_GeneCtrl[[i]]$Expres$ERBB2 >
+                         GMM_Sets[[i]]$ERBB2$Cut_off, 1,
+                       ifelse(VAR_GeneCtrl[[i]]$Expres$ERBB2 <=
+                                GMM_Sets[[i]]$ERBB2$Cut_off, 0, NA )) ))
+ }
> VAR_Gene <- data.frame(VAR_Gene)
```

## 2.2 GES Classifications & metagenes, 32 data sets

```
> RES_List <- list()
> for ( i in 1:nrow(Set)){
+   load(paste(Path, "RData_ObjSets", Set$File_Name[i], sep="/"))
+   #### import chip
+   load(paste(Path, "/RData_ObjSets/chip/", annotation(Eset_Obj), ".RData", sep=""))
+   CHIP <- CHIP[match(featureNames(Eset_Obj), CHIP$PID),]  # ctrl
+   #### data matrix
+   DATA <- exprs(Eset_Obj)
+   #### select samples set
+   sel_sampleloop <- which(PublicSet$HC$SetName %in% Set$Nom_ObjHC[i])
+   ################################
+   ##### Classification
+   ##### PAM50 & ROR_P :
+   source("g:/Ori Tools/Prog/Rwork/Script/Classifier/20100929_Classif.SSP_PAM50.r")
+   PAM50_Tmp <- PAM50.PF(DATA, CHIP$GeneID, Set$Nom_ObjHC[i])
+   RES_PAM50_Tmp <- cbind(PAM50_Tmp[,6:12])
+   ##### CLDN-low (Prat et al.) :
+   source("g:/Ori Tools/Prog/Rwork/Script/Classifier/20101123_Classif.Claudin_Low_PratBCL.r")
+   CLDN_Tmp <- Claudin_Low.Classifier(DATA, CHIP$GeneID, Set$Nom_ObjHC[i])
+   RES_CLDN_Tmp <- CLDN_Tmp$Grp
+   ##### Differentiation Score (Prat et al. /w Lum Prog., MaSC & Mat. Lum Lim et al.) :
+   source("g:/Ori Tools/Prog/Rwork/Script/Classifier/20101203_Classif.Diff_Prat.r")
+   DiffPrat_Tmp <- Diff_Prat_Classifier(DATA, CHIP, Set$Nom_ObjHC[i])
+   RES_DiffPrat_Tmp <- DiffPrat_Tmp
+   ##### Amsterdam 70g (vant Veer et al.)
+   source("g:/Ori Tools/Prog/Rwork/Script/Classifier/20100922_Classif.70g_vVeer.r")
+   vV70g_Tmp <- vV70g.Classifier(DATA, CHIP$GeneID, Set$Nom_ObjHC[i])
+   RES_vV70g_Tmp <- vV70g_Tmp$Classif
+   ##### GGI (Loi et al.)
+   source("g:/Ori Tools/Prog/Rwork/Script/Classifier/20100720_GGI.r")
+   GGI_Tmp <- GGI.Classifier(DATA, CHIP$GeneID,
+                             Grd=PublicSet$HC$Grade[sel_sampleloop],
+                             Set$Nom_ObjHC[i])
+   RES_GGI_Tmp <- GGI_Tmp$Classif[,1:2]
+   ##### Recurent Score (Paik et al.)
+   source("g:/Ori Tools/Prog/Rwork/Script/Classifier/20100922_Classif.RS.Paik.R")
+   RSPaik_Tmp <- RS.Paik.Classifier(DATA, CHIP$GeneID, Set$Nom_ObjHC[i])
+   RES_RSPaik_Tmp <- RSPaik_Tmp$Classif
+   ##### Immune Response (Caldas et al.)
+   source("g:/Ori Tools/Prog/Rwork/Script/Classifier/20121128_Classif.IR.Caldas.r")
+   IR_Tmp <- IR.Caldas_PF(DATA, CHIP, ER=VAR_Gene$ESR1[sel_sampleloop],
+                          Set$Nom_ObjHC[i])
+   RES_IR_Tmp <- data.frame(IR_Tmp)
+   ##### LCK (Rody et al.)
+   source("g:/Ori Tools/Prog/Rwork/Script/Classifier/20121129_Classif.LCK.Rody.r")
+   LCK_Tmp <- LCK.Rody_PF(DATA, CHIP, Set$Nom_ObjHC[i])
+   RES_LCK_Tmp <- data.frame(LCK_Tmp)
+   ##### Stroma (Bianchini et al.)
+   source("g:/Ori Tools/Prog/Rwork/Script/Classifier/20101007_Classif.Stromal_Bianchini.r")
+   StromaBianchini_Tmp <- Bianchini_Stroma.Classifier(DATA,
+                                 SSP = PAM50_Tmp$PAM50 , CHIP$GeneID, Set$Nom_ObjHC[i])
+   RES_StromaBianchini_Tmp <- StromaBianchini_Tmp$Classif
+   ##### Immune28g (Sabatier et al.)
+   source("g:/Ori Tools/Prog/Rwork/Script/Classifier/20111214_Classif.K.Imm_28g_RS.r")
+   Kimm28g_Tmp <- K.Imm_28g.Classifier(DATA, CHIP$GeneID,
+                                   SSP = PAM50_Tmp$PAM50, Set$Nom_ObjHC[i])
+   RES_Kimm28g_Tmp <- Kimm28g_Tmp$Classif[,1:2]
```

```
+   ##### Lympho metagenes (Palmer et al.)
+   source("g:/Ori Tools/Prog/Rwork/Script/Classifier/20130312_Palmer_MetaG_Lympho.r")
+   Palmer_Tmp <- Palmer_Lympho.PF(DATA, CHIP$GeneID, Set$Nom_ObjHC[i])
+   colnames(Palmer_Tmp) <- paste("Palmer", colnames(Palmer_Tmp), "Module", sep="_")
+   ##### DLDA30 (Hess et al.)
+   source("g:/Ori Tools/Prog/Rwork/Script/Classifier/20120905_Classif.DLDA_30_Hess.r")
+   DLDA30_Tmp <- DLDA30.Classifier_PF(DATA, CHIP$GeneID, Set$Nom_ObjHC[i])
+   RES_DLDA30_Tmp <- data.frame(DLDA30_Tmp$Classif)
+   ##### DCN (Farmer et al.)
+   source("g:/Ori Tools/Prog/Rwork/Script/Classifier/20120906_Classif.DCN.Farmer.r")
+   DCN_Tmp <- DCN.Classifier_PF(DATA, CHIP, Set$Nom_ObjHC[i], ER=VAR_Gene$ESR1[sel_sampleloop])
+   RES_DCN_Tmp <- data.frame(DCN_Tmp$Classif)
+   #### A_Score (Desmedt et al.)
+   source("g:/Ori Tools/Prog/Rwork/Script/Classifier/20120907_Classif.A_Score.Desmedt2k11.r")
+   A_Score_Tmp <- A_Score.Classifier_PF(DATA, CHIP, Set$Nom_ObjHC[i],
+                                        ER=VAR_Gene$ESR1[sel_sampleloop], ESR1_UNC=TRUE)
+   RES_A_Score_Tmp <- data.frame(A_Score_Tmp$Classif)
+   ##### RBloss Ertel
+   source("g:/Ori Tools/Prog/Rwork/Script/Classifier/20121129_Classif.RB_Ertel.r")
+   RBloss_Tmp <- RBloss.Ertel_PF(DATA, CHIP, ER=VAR_Gene$ESR1[sel_sampleloop],
+                                 SSP=PAM50_Tmp$PAM50 , Set$Nom_ObjHC[i])
+   RES_RBloss_Tmp <- data.frame(RBloss_Tmp[1:3])
+   ##### Taube EMT core
+   source("g:/Ori Tools/Prog/Rwork/Script/Classifier/20140606_Taube_EMT_MetaG.r")
+   RES_Taube_Tmp <- Taube_EM.PF(DATA, CHIP$GeneID, Set$Nom_ObjHC[i])
+   ##### Lum & Prolif cluster metagene, CH IPC BC
+   source("g:/Ori Tools/Prog/Rwork/Script/Classifier/20140106_MetaG_Lum_Prolif_CH353IPC.r")
+   MetaG_CH_IPC_Tmp <- MetaG_LumProlif.PF(DATA, CHIP$GeneID, Set$Nom_ObjHC[i])
+   ###########################
+   ##### Pool GESs
+   RES_Glob_Tmp <- cbind( RES_PAM50_Tmp, RES_CLDN_Tmp, RES_DiffPrat_Tmp,
+                          RES_vV70g_Tmp, RES_GGI_Tmp, RES_RSPaik_Tmp,
+                          RES_IR_Tmp, RES_LCK_Tmp, RES_StromaBianchini_Tmp,
+                          RES_Kimm28g_Tmp, RES_DLDA30_Tmp, RES_DCN_Tmp,
+                          RES_A_Score_Tmp, RES_RBloss_Tmp, RES_Taube_Tmp,
+                          MetaG_CH_IPC_Tmp )
+   RES_List[[i]] <- RES_Glob_Tmp
+   rm(CHIP, DATA, Eset_Obj)
+ }
> ##### Merge Classification
> RES_Pool <- c()
> for (i in 1:length(RES_List)){
+     RES_Pool <- rbind(RES_Pool, cbind(RES_List[[i]],
+                                 Set=rep(Set$Nom_ObjHC[i], nrow(RES_List[[i]]))))
+ }
> colnames(RES_Pool) <-paste("GES", colnames(RES_Pool), sep="_")
```

Variables preparation before statistics:

```
> ###### standardization Gene Ctrl / PAM50
> VAR_GeneSTDz <- c()
> for (i in seq_along(VAR_GeneCtrl)){
+   VAR_GeneSTDz <- rbind(data.frame(
+     SSPNorm_IPC_PF(VAR_GeneCtrl[[i]], SSP=RES_List[[i]]$PAM50)$EXPRS$NORM))
+ }
> VAR_GeneSTDz <- data.frame(VAR_GeneSTDz)
> colnames(VAR_GeneSTDz) <- paste("mRNA", colnames(VAR_GeneSTDz), "RAW", sep="_")
> colnames(VAR_Gene) <- paste("mRNA", colnames(VAR_Gene), sep="_")
> ###### pool all variables
```

```
> VAR_List <- c(as.list(PublicSet$HC),
+               as.list(VAR_GeneSTDz),
+               as.list(VAR_Gene),
+               as.list(RES_Pool))
> VAR_ListSurv <- list(DFS =
+                        list(Evt=PublicSet$HC$DFS, Del=PublicSet$HC$DFSDel))
```

# 3 Statistics

## 3.1 Description PAM50/CLDN-low BC samples

```
> #### PAM50 /w CLDN-low
> VAR_List$PAM50_CL <- ifelse(VAR_List$GES_CLDNLow_Grpe == "Claudin_Low", "CLDNlow",
+                             as.character(VAR_List$GES_PAM50))
> #### TN expression
> VAR_List$TN <- rep(NA, length(VAR_List[[1]]))
> VAR_List$TN[which(VAR_List$mRNA_ESR1==0 &
+                   VAR_List$mRNA_PGR==0 &
+                   VAR_List$mRNA_ERBB2==0)] <- "yes"
> VAR_List$TN[which(VAR_List$mRNA_ESR1==1 |
+                   VAR_List$mRNA_PGR==1 |
+                   VAR_List$mRNA_ERBB2==1)] <- "no"
```

### 3.1.1 PAM50/CLDNlow, clinicopathological features

```
> source("g:/Ori Tools/Prog/Rwork/Script/Table.PF/Table.PF.r")
> print(xtable(
+   Table_PF(VAR_List$PAM50_CL,
+              list(Age=VAR_List$age.g,
+             Histo_type=VAR_List$Histo_OK,
+             Histo_grade=VAR_List$Grade,
+             pT=VAR_List$pT2K,
+             pN=VAR_List$pN,
+             ESR1_groups=VAR_List$mRNA_ESR1,
+             ESR1_cont = VAR_List$mRNA_ESR1_RAW,
+             PGR_groups=VAR_List$mRNA_PGR,
+             PGR_cont = VAR_List$mRNA_PGR_RAW,
+             ERBB2_groups=VAR_List$mRNA_ERBB2,
+             ERBB2_cont = VAR_List$mRNA_ERBB2_RAW,
+             TN_exp=VAR_List$TN,
+             pCR=VAR_List$response.CT.pCR.no.invasive.cancer.,
+             DFS=VAR_ListSurv$DFS$Evt),
+        Var_Cont=c(7,9,11), Subset=sel_Primaire)[,c(1:3,5,7,4,6,8,9)]
+     , caption="Description PAM50/CLDNlow BCs", table.placement="top"), size="tiny")
```

| | Var | Mod | X3 | CLDNlow | LumA | Basal | ERBB2 | LumB | Normal |
|---|---|---|---|---|---|---|---|---|---|
| 1 | Age | | | | | | | | |
| 2 | | <=50 | 1834 | 238(49%) | 470(43%) | 423(56%) | 224(43%) | 328(46%) | 151(53%) |
| 3 | | >50 | 2005 | 247(51%) | 622(57%) | 331(44%) | 291(57%) | 380(54%) | 134(47%) |
| 4 | Histo_type | | | | | | | | |
| 5 | | IDC | 1181 | 140(78%) | 263(76%) | 224(88%) | 201(89%) | 255(89%) | 98(84%) |
| 6 | | ILC | 72 | 8(4%) | 34(10%) | 4(2%) | 4(2%) | 12(4%) | 10(9%) |
| 7 | | MED | 24 | 5(3%) | 1(0%) | 18(7%) | 0(0%) | 0(0%) | 0(0%) |
| 8 | | MIX | 59 | 7(4%) | 23(7%) | 4(2%) | 10(4%) | 12(4%) | 3(3%) |
| 9 | | other | 77 | 20(11%) | 26(7%) | 6(2%) | 11(5%) | 8(3%) | 6(5%) |
| 10 | Histo_grade | | | | | | | | |
| 11 | | 1 | 489 | 49(9%) | 293(26%) | 12(2%) | 15(3%) | 53(7%) | 67(21%) |
| 12 | | 2 | 1579 | 180(35%) | 605(54%) | 104(14%) | 170(32%) | 367(47%) | 153(48%) |
| 13 | | 3 | 1957 | 290(56%) | 222(20%) | 640(85%) | 350(65%) | 358(46%) | 97(31%) |
| 14 | pT | | | | | | | | |
| 15 | | pT1 | 928 | 106(38%) | 343(46%) | 130(29%) | 89(27%) | 163(34%) | 97(50%) |
| 16 | | pT2-3 | 1542 | 172(62%) | 399(54%) | 323(71%) | 239(73%) | 311(66%) | 98(50%) |
| 17 | pN | | | | | | | | |
| 18 | | 0 | 1941 | 182(55%) | 561(62%) | 386(67%) | 236(52%) | 395(61%) | 181(60%) |
| 19 | | 1 | 1279 | 151(45%) | 344(38%) | 193(33%) | 220(48%) | 250(39%) | 121(40%) |
| 20 | ESR1_groups | | | | | | | | |
| 21 | | 0 | 1929 | 433(64%) | 80(5%) | 859(86%) | 437(58%) | 24(2%) | 96(21%) |
| 22 | | 1 | 3518 | 240(36%) | 1414(95%) | 144(14%) | 312(42%) | 1053(98%) | 355(79%) |
| 23 | ESR1_cont | | 5447 | 6.46(-4.93-12.71) | 10.55(-2.06-14.78) | 5.46(-5.41-13.44) | 7.18(-4.98-14.42) | 10.76(-1.92-14.85) | 9.77(-2.75-14.43) |
| 24 | PGR_groups | | | | | | | | |
| 25 | | 0 | 2851 | 445(66%) | 386(26%) | 864(86%) | 567(76%) | 443(41%) | 146(32%) |
| 26 | | 1 | 2594 | 228(34%) | 1108(74%) | 139(14%) | 181(24%) | 634(59%) | 304(68%) |
| 27 | PGR_cont | | 5445 | 4.21(-2.86-11.59) | 5.24(-3.45-12.96) | 3.54(-6.69-11.13) | 4.07(-4.89-12.18) | 4.52(-3.06-13.09) | 5.07(-3.22-12.38) |
| 28 | ERBB2_groups | | | | | | | | |
| 29 | | 0 | 4738 | 646(96%) | 1407(94%) | 958(96%) | 320(43%) | 1011(94%) | 396(88%) |
| 30 | | 1 | 709 | 27(4%) | 87(6%) | 45(4%) | 429(57%) | 66(6%) | 55(12%) |
| 31 | ERBB2_cont | | 5447 | 6.45(-3.95-12.58) | 7.5(-2.84-14.27) | 6.52(-4.03-13.37) | 8.84(-2.41-15.61) | 7.59(-3.67-14.19) | 7.83(-2.97-15.53) |
| 32 | TN_exp | | | | | | | | |
| 33 | | no | 4110 | 321(48%) | 1472(99%) | 241(24%) | 610(82%) | 1064(99%) | 402(89%) |
| 34 | | yes | 1336 | 352(52%) | 22(1%) | 762(76%) | 138(18%) | 13(1%) | 49(11%) |
| 35 | pCR | | | | | | | | |
| 36 | | nPCR | 992 | 155(68%) | 302(93%) | 210(67%) | 97(63%) | 178(82%) | 50(86%) |
| 37 | | pCR | 302 | 73(32%) | 21(7%) | 104(33%) | 56(37%) | 40(18%) | 8(14%) |
| 38 | DFS | | | | | | | | |
| 39 | | 0 | 2190 | 223(65%) | 736(75%) | 396(62%) | 222(52%) | 395(60%) | 218(73%) |
| 40 | | 1 | 1165 | 120(35%) | 246(25%) | 245(38%) | 205(48%) | 268(40%) | 81(27%) |

Description PAM50/CLDNlow BCs

#### 3.1.2 PAM50/CLDNlow, survival description (DFS)

```
> source("g:/Ori Tools/Prog/Rwork/Script/survie.PF/Survival.PF/R/Surv.PF-28072014.r")
> source("g:/Ori Tools/Prog/Rwork/Script/survie.PF/Survival.PF/R/PlotSurv.PF.R")
> #### DFS all
> KM_DFS <- Surv.PF(NULL, VAR_ListSurv$DFS$Evt, VAR_ListSurv$DFS$Del, "Disease-free",
+                       Subset=sel_Primaire)

 Disease-free survival

Call: survfit(formula = Surv(EvtDel, Evt) ~ rep(1, length(Evt)), subset = Subset)

   2092 observations deleted due to missingness
records   n.max n.start  events   median 0.95LCL 0.95UCL
   3355    3355    3355    1165      171     152      NA

N=3355,
5-Year  survival = 68%  (95CI = [0.67-0.7])
50% survival time (month) : 171.4
Follow-up median (months) : 79

> #### DFS CLDNlow
> KM_DFS_CL <- Surv.PF(NULL, VAR_ListSurv$DFS$Evt, VAR_ListSurv$DFS$Del, "Disease-free",
+                       Subset=intersect(sel_Primaire,which(VAR_List$PAM50_CL %in% "CLDNlow")))

 Disease-free survival

Call: survfit(formula = Surv(EvtDel, Evt) ~ rep(1, length(Evt)), subset = Subset)

   330 observations deleted due to missingness
records   n.max n.start  events   median 0.95LCL 0.95UCL
    343     343     343     120      141     140      NA

N=343,
5-Year  survival = 67%  (95CI = [0.62-0.73])
```

```
50% survival time (month) : 141
Follow-up median (months) : 72.24

> #### DFS PAM50/CLDNlow
> KM_DFS_PAM50CL <- Surv.PF(VAR_List$PAM50_CL, VAR_ListSurv$DFS$Evt, VAR_ListSurv$DFS$Del,
+                           "Disease-free", Subset=sel_Primaire)

 Disease-free survival

Call:
survdiff(formula = Surv(EvtDel, Evt) ~ Grpe, subset = Subset)

n=3355, 2092 observations deleted due to missingness.

               N Observed Expected (O-E)^2/E (O-E)^2/V
Grpe=Basal   641      245      186    18.487    22.084
Grpe=CLDNlow 343      120      111     0.777     0.861
Grpe=ERBB2   427      205      133    38.432    43.504
Grpe=LumA    982      246      381    48.072    71.685
Grpe=LumB    663      268      231     5.999     7.500
Grpe=Normal  299       81      122    13.986    15.679

 Chisq= 126  on 5 degrees of freedom, p= 0

Grp 1 : Basal N=641, 5-Year survival = 60%, 95%CI = [0.56-0.64]
Grp 2 : CLDNlow N=343, 5-Year survival = 67%, 95%CI = [0.62-0.73]
Grp 3 : ERBB2 N=427, 5-Year survival = 55%, 95%CI = [0.5-0.6]
Grp 4 : LumA N=982, 5-Year survival = 80%, 95%CI = [0.77-0.83]
Grp 5 : LumB N=663, 5-Year survival = 64%, 95%CI = [0.6-0.68]
Grp 6 : Normal N=299, 5-Year survival = 79%, 95%CI = [0.75-0.84]

> #### Kaplan-Meier plot, DFS PAM50/CL
> col_PAM50CL <- c("red","orange","magenta","blue","cyan","green")
> PlotSurv.PF(KM_DFS_PAM50CL, limit=c(0,120), mark=FALSE, style=1,
+             color = col_PAM50CL, texte = FALSE, pVal = FALSE)
> legend("bottomleft", sort(unique(VAR_List$PAM50_CL)), col=col_PAM50CL, lwd=3, box.col=0, cex=0.75)
```

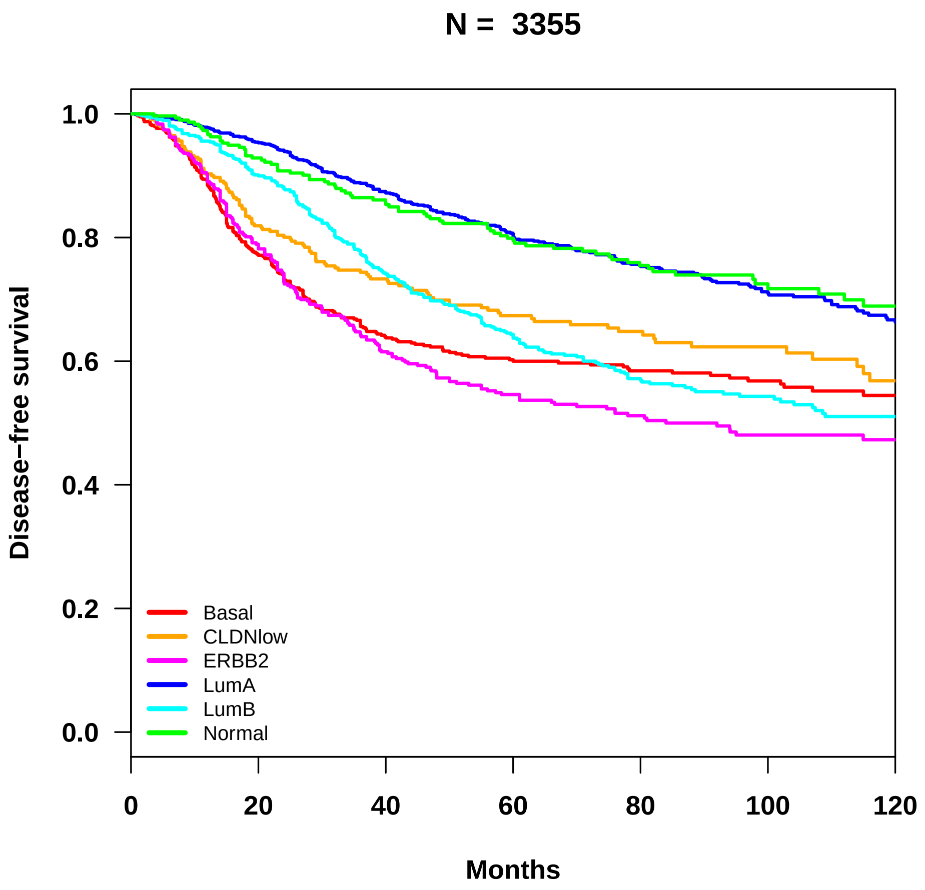


```
> #### Freq. relapse f(t), Claudin-low, Basal & LuminalA BC
> source("g:/Ori Tools/Prog/Rwork/Script/survie.PF/Survival.PF/R/FreqRelapse.PF.r")
> Freq_3KPAM50CLDN <-FreqSurv_PF(VAR_List$PAM50_CL,
+                               VAR_ListSurv$DFS$Evt,
+                               VAR_ListSurv$DFS$Del,
+                               Subset=intersect(sel_Primaire,
+                                                which(VAR_List$PAM50_CL %in%
+                                                        c("Basal", "CLDNlow", "LumA"))))
> #### Plot Freq. relapse f(t), 3 moleclar subtypes
> Plot_FreqRelapse.PF(Freq_3KPAM50CLDN,
+                     col= col_PAM50CL[c(1,2,4)], xlim=c(0,250),
+                     ylim=c(0,0.18), lwd=3)
> legend("topright", sort(unique(VAR_List$PAM50_CL))[c(1,2,4)],
+        col=col_PAM50CL[c(1,2,4)],
+        lwd=3, box.col=0, cex=0.75)
```

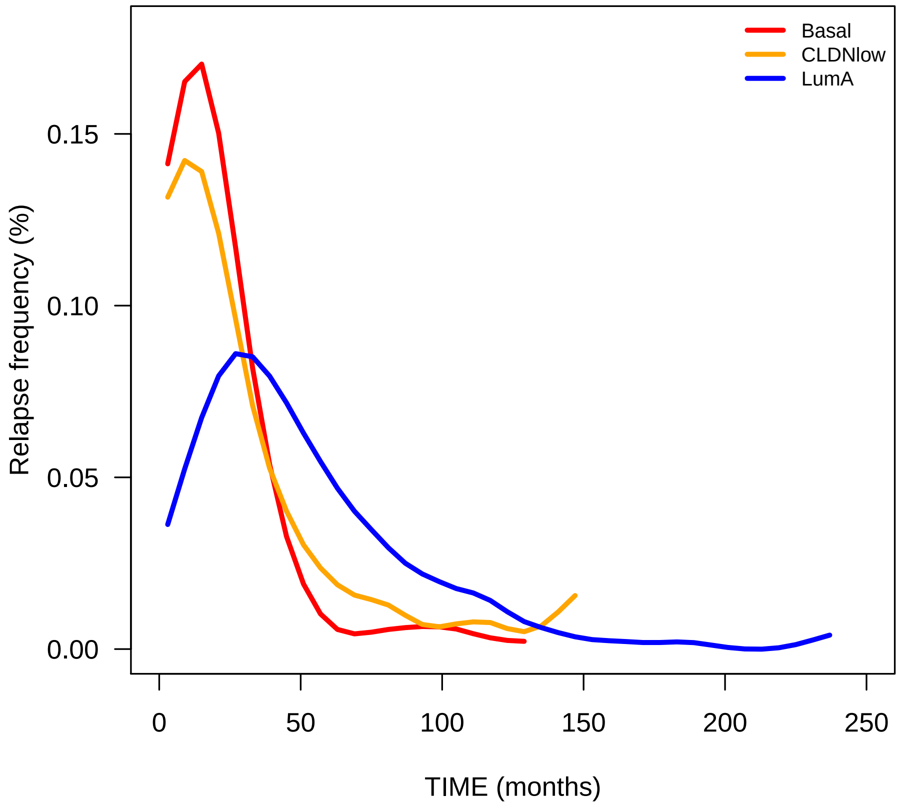


### 3.2 Pronostic associations

Variables evaluation, univariate DFS

```
> ###### coxph regression for batch run (use of survival R package)
> source("g:/Ori Tools/Prog/Rwork/Script/20121008_UV.batch.r")
> #### All primary BC
> UV_DFS_AllBC <- Batch.UV.PF(List.Grpe = list(Age=VAR_List$age.g,
+                                          Histo_type=VAR_List$Histo_OK,
+                                          Histo_grade=VAR_List$Grade2K,
+                                          pT=VAR_List$pT2K,
+                                          pN=VAR_List$pN,
+                                          ESR1_groups=VAR_List$mRNA_ESR1,
+                                          PGR_groups=VAR_List$mRNA_PGR,
+                                          ERBB2_groups=VAR_List$mRNA_ERBB2,
+                                          TN_exp=VAR_List$TN,
+                                          Amst70g=VAR_List$GES_Prog70g_Grp,
+                                          GGI=VAR_List$GES_GGI_Grp,
+                                          RS=VAR_List$GES_RSPaik_Grp,
+                                          ROR_P=VAR_List$GES_PAM50_ROR_P_Grp,
+                                          IR=VAR_List$GES_IR_Caldas_IR_GrpERdep,
+                                          LCK=VAR_List$GES_LCK_Rody_LCK_Grp,
+                                          ImmuneKin28g = VAR_List$GES_K.Imm28g_Grp,
+                                          B_cellK = VAR_List$GES_StromaBianchini_metagene_Terc),
+                          Evt = VAR_ListSurv$DFS$Evt, EvtDel = VAR_ListSurv$DFS$Del,
+                          Subset = sel_Primaire, PRINT = FALSE)
> # Print global UV table
```

```
> print(xtable(UV_DFS_AllBC$Res.Batch,
+              caption="univariate DFS, all BC", table.placement="top"),
+       size="footnotesize")
```

| | VAR | MODTEST | n | HR.95CI. | p |
|---|---|---|---|---|---|
| 1 | Age | >50 | 2366 | 1.01 [0.87-1.16] | 0.922 |
| 2 | Histo_type | ILC | 624 | 1.09 [0.71-1.67] | 0.269 |
| 3 | | MED | | 0.53 [0.20-1.42] | |
| 4 | | MIX | | 0.44 [0.18-1.06] | |
| 5 | | other | | 1.04 [0.57-1.92] | |
| 6 | Histo_grade | 2-3 | 2552 | 2.36 [1.86-3.01] | 2.84e-12 |
| 7 | pT | pT2-3 | 1744 | 1.52 [1.29-1.8] | 6.86e-07 |
| 8 | pN | 1 | 2366 | 1.37 [1.18-1.58] | 2.28e-05 |
| 9 | ESR1_groups | 1 | 3355 | 0.56 [0.5-0.63] | 0 |
| 10 | PGR_groups | 1 | 3353 | 0.7 [0.63-0.79] | 2.25e-09 |
| 11 | ERBB2_groups | 1 | 3355 | 1.4 [1.18-1.65] | 7.32e-05 |
| 12 | TN_exp | yes | 3354 | 1.7 [1.49-1.94] | 1.11e-15 |
| 13 | Amst70g | Poor | 3355 | 1.83 [1.57-2.12] | 3e-15 |
| 14 | GGI | High | 2655 | 2.1 [1.82-2.43] | 0 |
| 15 | RS | High | 3355 | 2.15 [1.87-2.47] | 0 |
| 16 | | Intermediate | | 1.63 [1.38-1.93] | |
| 17 | ROR_P | High | 3355 | 2.11 [1.85-2.41] | 0 |
| 18 | | Median | | 1.75 [1.45-2.10] | |
| 19 | IR | Poor | 3354 | 1.19 [1.06-1.34] | 0.00292 |
| 20 | LCK | High | 3355 | 0.91 [0.79-1.04] | 0.151 |
| 21 | ImmuneKin28g | Poor | 3355 | 1.04 [0.88-1.24] | 0.615 |
| 22 | B_cellK | 2_Int | 3353 | 0.87 [0.76-1.00] | 3.94e-05 |
| 23 | | 3_High | | 0.72 [0.63-0.83] | |

univariate DFS, all BC

```
> #### PAM50/CL BC
> Subtype <- sort(unique(VAR_List$PAM50_CL))
> UV_DFS_PAM50CL <- list()
> for (i in seq_along(Subtype)){
+   UV_DFS_PAM50CL[[i]] <- Batch.UV.PF(List.Grpe = list(Age=VAR_List$age.g,
+                                       Histo_type=VAR_List$Histo_OK,
+                                       Histo_grade=VAR_List$Grade2K,
+                                       pT=VAR_List$pT2K,
+                                       pN=VAR_List$pN,
+                                       ESR1_groups=VAR_List$mRNA_ESR1,
+                                       PGR_groups=VAR_List$mRNA_PGR,
+                                       ERBB2_groups=VAR_List$mRNA_ERBB2,
+                                       TN_exp=VAR_List$TN,
+                                       Amst70g=VAR_List$GES_Prog70g_Grp,
+                                       GGI=VAR_List$GES_GGI_Grp,
+                                       RS=VAR_List$GES_RSPaik_Grp,
+                                       ROR_P=VAR_List$GES_PAM50_ROR_P_Grp,
+                                       IR=VAR_List$GES_IR_Caldas_IR_GrpERdep,
+                                       LCK=VAR_List$GES_LCK_Rody_LCK_Grp,
+                                       ImmuneKin28g = VAR_List$GES_K.Imm28g_Grp,
+                                       B_cellK = VAR_List$GES_StromaBianchini_metagene_Terc),
+                        Evt = VAR_ListSurv$DFS$Evt, EvtDel = VAR_ListSurv$DFS$Del,
+                        Subset = intersect(sel_Primaire, which(VAR_List$PAM50_CL %in% Subtype[i])),
+                        PRINT = FALSE)
+   names(UV_DFS_PAM50CL)[i] <- Subtype[i]
+ }
> # Print CLDN UV table
> print(xtable(UV_DFS_PAM50CL$CLDNlow$Res.Batch,
+              caption="univariate DFS, CLDN-low", table.placement="top"),
+       size="footnotesize")
```

| | VAR | MODTEST | n | HR.95CI. | p |
|---|---|---|---|---|---|
| 1 | Age | >50 | 237 | 0.9 [0.57-1.4] | 0.636 |
| 2 | Histo_type | ILC | 59 | 0.00 [0.00- Inf] | 0.837 |
| 3 | | MED | | 0.45 [0.06-3.35] | |
| 4 | | MIX | | 0.00 [0.00- Inf] | |
| 5 | | other | | 1.90 [0.44-8.10] | |
| 6 | Histo_grade | 2-3 | 270 | 3.63 [1.32-9.92] | 0.0122 |
| 7 | pT | pT2-3 | 155 | 2.33 [1.27-4.26] | 0.00601 |
| 8 | pN | 1 | 194 | 2.23 [1.34-3.72] | 0.00201 |
| 9 | ESR1_groups | 1 | 343 | 0.38 [0.25-0.58] | 5.02e-06 |
| 10 | PGR_groups | 1 | 343 | 0.56 [0.38-0.83] | 0.00386 |
| 11 | ERBB2_groups | 1 | 343 | 1.98 [0.92-4.26] | 0.079 |
| 12 | TN_exp | yes | 343 | 2.19 [1.51-3.18] | 3.82e-05 |
| 13 | Amst70g | Poor | 343 | 1.22 [0.74-2.02] | 0.435 |
| 14 | GGI | High | 283 | 1.42 [0.92-2.2] | 0.113 |
| 15 | RS | High | 343 | 3.03 [1.68-5.46] | 0.00109 |
| 16 | | Intermediate | | 2.73 [1.40-5.31] | |
| 17 | ROR_P | High | 343 | 1.85 [1.24-2.75] | 0.00874 |
| 18 | | Median | | 1.28 [0.69-2.36] | |
| 19 | IR | Poor | 343 | 1.17 [0.82-1.68] | 0.387 |
| 20 | LCK | High | 343 | 0.89 [0.62-1.28] | 0.541 |
| 21 | ImmuneKin28g | Poor | 343 | 1.14 [0.78-1.65] | 0.507 |
| 22 | B_cellK | 2_Int | 343 | 0.70 [0.44-1.11] | 0.0692 |
| 23 | | 3_High | | 0.61 [0.40-0.93] | |

univariate DFS, CLDN-low

## 3.3 Pathological response associations

Evaluation pCR and clinicopathological&molecular features

```
> #### All primary BC
> TabpCR_AllBC <- Table_PF(VAR_List$response.CT.pCR.no.invasive.cancer.,
+                          Var = list(Age=VAR_List$age.g, pT=VAR_List$pT2K,
+                                     Histo_grade=VAR_List$Grade2K, Histo_type=VAR_List$Histo_OK,
+                                     ESR1_groups=VAR_List$mRNA_ESR1, PGR_groups=VAR_List$mRNA_PGR,
+                                     ERBB2_groups=VAR_List$mRNA_ERBB2, TN_exp=VAR_List$TN,
+                                     DLDA30=VAR_List$GES_DLDA30Hess_GrpTxt,
+                                     Stromal=VAR_List$GES_DCNFarmer_grp,
+                                     A_Score=VAR_List$GES_A_ScoreDesmedt_grp,
+                                     RBloss=VAR_List$GES_RBloss_Ertel_RB_Grp2K),
+                         Subset = sel_Primaire, Print = FALSE)
> # Print global pCR table
> print(xtable(TabpCR_AllBC, caption="pCR, all BC", table.placement="!h"),
+       size="scriptsize")
```

| | Var | Mod | X3 | nPCR | pCR | p..NonParam | Statistic |
|---|---|---|---|---|---|---|---|
| 1 | Age | | | | | 0.0868 | 0.8 |
| 2 | | <=50 | 697 | 521(53%) | 176(58%) | | [0.61-1.04] |
| 3 | | >50 | 595 | 469(47%) | 126(42%) | | |
| 4 | pT | | | | | 0.859 | 1.1 |
| 5 | | pT1 | 44 | 32(14%) | 12(13%) | | [0.53-2.53] |
| 6 | | pT2-3 | 279 | 196(86%) | 83(87%) | | |
| 7 | Histo_grade | | | | | 0.000168 | 8.1 |
| 8 | | 1 | 53 | 51(6%) | 2(1%) | | [2.11-69.38] |
| 9 | | 2-3 | 1126 | 854(94%) | 272(99%) | | |
| 10 | Histo_type | | | | | 0.752 | |
| 11 | | IDC | 468 | 337(82%) | 131(85%) | | |
| 12 | | ILC | 15 | 10(2%) | 5(3%) | | |
| 13 | | MIX | 37 | 29(7%) | 8(5%) | | |
| 14 | | other | 47 | 36(9%) | 11(7%) | | |
| 15 | ESR1_groups | | | | | 7.21e-18 | 0.3 |
| 16 | | 0 | 697 | 470(47%) | 227(75%) | | [0.22-0.4] |
| 17 | | 1 | 597 | 522(53%) | 75(25%) | | |
| 18 | PGR_groups | | | | | 4.24e-08 | 0.38 |
| 19 | | 0 | 979 | 716(72%) | 263(87%) | | [0.26-0.56] |
| 20 | | 1 | 315 | 276(28%) | 39(13%) | | |
| 21 | ERBB2_groups | | | | | 0.00059 | 1.9 |
| 22 | | 0 | 1125 | 881(89%) | 244(81%) | | [1.31-2.7] |
| 23 | | 1 | 169 | 111(11%) | 58(19%) | | |
| 24 | TN_exp | | | | | 8.16e-10 | 2.3 |
| 25 | | no | 777 | 642(65%) | 135(45%) | | [1.73-2.97] |
| 26 | | yes | 517 | 350(35%) | 167(55%) | | |
| 27 | DLDA30 | | | | | 7.53e-23 | 0.26 |
| 28 | | pCR_like | 439 | 264(27%) | 175(58%) | | [0.2-0.35] |
| 29 | | RD_like | 855 | 728(73%) | 127(42%) | | |
| 30 | Stromal | | | | | 0.115 | 0.81 |
| 31 | | pCR_Like | 642 | 480(48%) | 162(54%) | | [0.62-1.06] |
| 32 | | RD_Like | 652 | 512(52%) | 140(46%) | | |
| 33 | A_Score | | | | | 0.000148 | 2 |
| 34 | | nonpCR_Like | 258 | 196(46%) | 62(30%) | | [1.36-2.85] |
| 35 | | pCR_Like | 378 | 233(54%) | 145(70%) | | |
| 36 | RBloss | | | | | 0.00652 | 1.7 |
| 37 | | Low | 1150 | 895(90%) | 255(84%) | | [1.14-2.51] |
| 38 | | High | 144 | 97(10%) | 47(16%) | | |

pCR, all BC

```
> #### PAM50/CL BC
> TabpCR_PAM50CL <- list()
> for (i in seq_along(Subtype)){
+   TabpCR_PAM50CL[[i]] <- Table_PF(VAR_List$response.CT.pCR.no.invasive.cancer.,
+                          Var = list(Age=VAR_List$age.g, pT=VAR_List$pT2K,
+                                     Histo_grade=VAR_List$Grade2K, Histo_type=VAR_List$Histo_OK,
+                                     ESR1_groups=VAR_List$mRNA_ESR1, PGR_groups=VAR_List$mRNA_PGR,
+                                     ERBB2_groups=VAR_List$mRNA_ERBB2, TN_exp=VAR_List$TN,
+                                     DLDA30=VAR_List$GES_DLDA30Hess_GrpTxt,
+                                     Stromal=VAR_List$GES_DCNFarmer_grp,
```

```
+                                      A_Score=VAR_List$GES_A_ScoreDesmedt_grp,
+                                      RBloss=VAR_List$GES_RBloss_Ertel_RB_Grp2K),
+                         Subset = intersect(sel_Primaire, which(VAR_List$PAM50_CL %in% Subtype[i])),
+                         Print = FALSE)
+   names(TabpCR_PAM50CL)[i] <- Subtype[i]
+ }
> # Print CLDN UV table
> print(xtable(TabpCR_PAM50CL$CLDNlow, caption="pCR, CLDN-low", table.placement="!h"),
+       size="scriptsize")
```

| | Var | Mod | X3 | nPCR | pCR | p..NonParam | Statistic |
|---|---|---|---|---|---|---|---|
| 1 | Age | | | | | 0.392 | 0.76 |
| 2 | | <=50 | 123 | 80(52%) | 43(59%) | | [0.41-1.37] |
| 3 | | >50 | 104 | 74(48%) | 30(41%) | | |
| 4 | pT | | | | | 1 | 0.93 |
| 5 | | pT1 | 5 | 3(8%) | 2(9%) | | [0.1-11.96] |
| 6 | | pT2-3 | 55 | 34(92%) | 21(91%) | | |
| 7 | Histo_grade | | | | | 0.0569 | Inf |
| 8 | | 1 | 8 | 8(6%) | 0(0%) | | [0.82-Inf] |
| 9 | | 2-3 | 204 | 136(94%) | 68(100%) | | |
| 10 | Histo_type | | | | | 0.125 | |
| 11 | | IDC | 77 | 45(67%) | 32(84%) | | |
| 12 | | ILC | 5 | 3(4%) | 2(5%) | | |
| 13 | | MIX | 6 | 6(9%) | 0(0%) | | |
| 14 | | other | 17 | 13(19%) | 4(11%) | | |
| 15 | ESR1_groups | | | | | 0.0735 | 0.47 |
| 16 | | 0 | 183 | 119(77%) | 64(88%) | | [0.19-1.07] |
| 17 | | 1 | 45 | 36(23%) | 9(12%) | | |
| 18 | PGR_groups | | | | | 0.42 | 0.64 |
| 19 | | 0 | 195 | 130(84%) | 65(89%) | | [0.24-1.57] |
| 20 | | 1 | 33 | 25(16%) | 8(11%) | | |
| 21 | ERBB2_groups | | | | | 0.472 | 1.7 |
| 22 | | 0 | 219 | 150(97%) | 69(95%) | | [0.33-8.34] |
| 23 | | 1 | 9 | 5(3%) | 4(5%) | | |
| 24 | TN_exp | | | | | 0.285 | 1.4 |
| 25 | | no | 71 | 52(34%) | 19(26%) | | [0.74-2.83] |
| 26 | | yes | 157 | 103(66%) | 54(74%) | | |
| 27 | DLDA30 | | | | | 0.0161 | 0.49 |
| 28 | | pCR_like | 120 | 73(47%) | 47(64%) | | [0.27-0.91] |
| 29 | | RD_like | 108 | 82(53%) | 26(36%) | | |
| 30 | Stromal | | | | | 0.623 | 1.2 |
| 31 | | pCR_Like | 55 | 39(25%) | 16(22%) | | [0.59-2.5] |
| 32 | | RD_Like | 173 | 116(75%) | 57(78%) | | |
| 33 | A_Score | | | | | 0.00315 | 3.9 |
| 34 | | nonpCR_Like | 37 | 31(32%) | 6(11%) | | [1.46-12.32] |
| 35 | | pCR_Like | 118 | 67(68%) | 51(89%) | | |
| 36 | RBloss | | | | | 1 | 0.96 |
| 37 | | Low | 212 | 144(93%) | 68(93%) | | [0.25-3.15] |
| 38 | | High | 16 | 11(7%) | 5(7%) | | |

pCR, CLDN-low

## 3.4 Correlation PAM50/CLDN-low and metagenes

List of metagenes

```
> MetaG_List <- list(Luminal=VAR_List$GES_MetaG_Lum_CH353IPC,
+       Prolif=VAR_List$GES_MetaG_Prolif_CH353IPC,
+       T_cells=VAR_List$GES_Palmer_T_Module,
+       B_cells=VAR_List$GES_Palmer_B_Module,
+       Granulocytes=VAR_List$GES_Palmer_GRANS_Module,
+       Core_EMT=VAR_List$GES_Taube_EMTMetaG_Score,
+       Differentiation_Score=VAR_List$GES_DiffPrat_Score,
+       Stem_cells_GES=VAR_List$GES_Creighton_CD44pCD24m_Score)
```

Plot Metagene ~ PAM50/CLDNlow

```
> VAR_List$PAM50_CL[which(VAR_List$PAM50_CL %in% "CLDNlow")] <- " CLDNlow"
> par(mfrow=c(2,4), mar=c(3,3,4,1))
> for (i in seq_along(MetaG_List)){
+   boxplot(MetaG_List[[i]]~VAR_List$PAM50_CL,
+             subset=sel_Primaire,
+             col=col_PAM50CL[c(2,1,3:6)], las=1,
+             main=names(MetaG_List)[i], outline=FALSE)
+ }
```

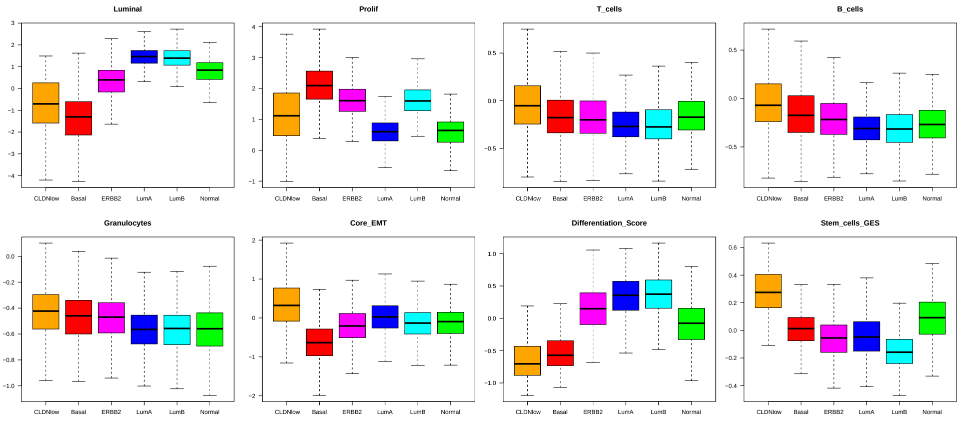


Metagenes : evaluation CLDNlow vs. others (TukeyHSD, ANOVA lm models)

```
> #### script format output aov(lm x ~ y) & TukeyHSD
> ## x : metagene x
> ## y : PAM50/CLDN-low
> source("G:\\Ori Tools\\Prog\\Rwork\\Script\\20130925_UVMV_glm.batch.r")
> UVGLM_PAM50CL <- list()
> for (i in seq_along(MetaG_List)){
+     UVGLM_PAM50CL[[i]] <- TukeyHSD_PF(lm(MetaG_List[[i]] ~ VAR_List$PAM50_CL,
+                                           subset=sel_Primaire), Ref="CLDNlow")
+     names(UVGLM_PAM50CL)[i] <- names(MetaG_List)[i]
+     xtmp <- xtable(UVGLM_PAM50CL[[i]],
+                   caption=gsub("_", "-", names(UVGLM_PAM50CL)[i]),
+                   table.placement="!h",
+                   display=c("f", "d", "f", "E", "E"))
+     print(xtmp, size="footnotesize", caption.placement="top")
+ }
```

Luminal

| | N | OddsRatio[CI95] | pvalue | p.adj |
|---|---|---|---|---|
| Basal vs CLDNlow | 5447 | 0.58[0.54-0.62] | 1.95E-43 | 5.85E-43 |
| ERBB2 vs CLDNlow | 5447 | 2.86[2.67-3.06] | 5.88E-133 | 3.53E-132 |
| LumA vs CLDNlow | 5447 | 8.73[8.23-9.27] | 0.00E+00 | 0.00E+00 |
| LumB vs CLDNlow | 5447 | 8.34[7.83-8.89] | 0.00E+00 | 0.00E+00 |
| Normal vs CLDNlow | 5447 | 4.27[3.95-4.62] | 2.89E-188 | 2.31E-187 |

Prolif

| | N | OddsRatio[CI95] | pvalue | p.adj |
|---|---|---|---|---|
| Basal vs CLDNlow | 5447 | 2.65[2.52-2.79] | 7.48E-195 | 8.23E-194 |
| ERBB2 vs CLDNlow | 5447 | 1.59[1.5-1.68] | 9.35E-43 | 2.81E-42 |
| LumA vs CLDNlow | 5447 | 0.54[0.52-0.57] | 2.86E-94 | 2.29E-93 |
| LumB vs CLDNlow | 5447 | 1.62[1.54-1.71] | 6.29E-54 | 2.51E-53 |
| Normal vs CLDNlow | 5447 | 0.55[0.51-0.58] | 3.64E-55 | 1.82E-54 |

T-cells

| | N | OddsRatio[CI95] | pvalue | p.adj |
|---|---|---|---|---|
| Basal vs CLDNlow | 5447 | 0.86[0.84-0.88] | 1.60E-27 | 2.08E-26 |
| ERBB2 vs CLDNlow | 5447 | 0.86[0.84-0.88] | 4.11E-26 | 4.93E-25 |
| LumA vs CLDNlow | 5447 | 0.8[0.78-0.81] | 5.62E-72 | 8.43E-71 |
| LumB vs CLDNlow | 5447 | 0.8[0.78-0.82] | 2.73E-61 | 3.82E-60 |
| Normal vs CLDNlow | 5447 | 0.87[0.85-0.89] | 3.09E-17 | 3.40E-16 |

B-cells

| | N | OddsRatio[CI95] | pvalue | p.adj |
|---|---|---|---|---|
| Basal vs CLDNlow | 5447 | 0.87[0.85-0.89] | 2.97E-25 | 2.67E-24 |
| ERBB2 vs CLDNlow | 5447 | 0.84[0.82-0.86] | 7.29E-36 | 8.01E-35 |
| LumA vs CLDNlow | 5447 | 0.75[0.74-0.77] | 2.29E-113 | 3.44E-112 |
| LumB vs CLDNlow | 5447 | 0.77[0.75-0.78] | 7.26E-91 | 1.02E-89 |
| Normal vs CLDNlow | 5447 | 0.79[0.77-0.81] | 1.04E-50 | 1.35E-49 |

Granulocytes

| | N | OddsRatio[CI95] | pvalue | p.adj |
|---|---|---|---|---|
| Basal vs CLDNlow | 5447 | 0.94[0.93-0.96] | 6.10E-09 | 3.05E-08 |
| ERBB2 vs CLDNlow | 5447 | 0.94[0.92-0.95] | 5.53E-10 | 3.32E-09 |
| LumA vs CLDNlow | 5447 | 0.86[0.85-0.87] | 1.70E-56 | 2.55E-55 |
| LumB vs CLDNlow | 5447 | 0.86[0.84-0.87] | 1.04E-53 | 1.46E-52 |
| Normal vs CLDNlow | 5447 | 0.85[0.83-0.87] | 1.47E-39 | 1.91E-38 |

Core-EMT

| | N | OddsRatio[CI95] | pvalue | p.adj |
|---|---|---|---|---|
| Basal vs CLDNlow | 5447 | 0.37[0.36-0.39] | 0.00E+00 | 0.00E+00 |
| ERBB2 vs CLDNlow | 5447 | 0.57[0.55-0.6] | 3.00E-99 | 3.60E-98 |
| LumA vs CLDNlow | 5447 | 0.71[0.68-0.74] | 7.29E-50 | 5.10E-49 |
| LumB vs CLDNlow | 5447 | 0.61[0.59-0.64] | 8.22E-89 | 9.04E-88 |
| Normal vs CLDNlow | 5447 | 0.61[0.58-0.64] | 1.02E-60 | 8.15E-60 |

Differentiation-Score

| | N | OddsRatio[CI95] | pvalue | p.adj |
|---|---|---|---|---|
| Basal vs CLDNlow | 5447 | 1.13[1.1-1.16] | 5.54E-14 | 1.11E-13 |
| ERBB2 vs CLDNlow | 5447 | 2.17[2.11-2.23] | 0.00E+00 | 0.00E+00 |
| LumA vs CLDNlow | 5447 | 2.62[2.56-2.69] | 0.00E+00 | 0.00E+00 |
| LumB vs CLDNlow | 5447 | 2.69[2.63-2.77] | 0.00E+00 | 0.00E+00 |
| Normal vs CLDNlow | 5447 | 1.71[1.66-1.77] | 5.50E-155 | 4.95E-154 |

Stem-cells-GES

| | N | OddsRatio[CI95] | pvalue | p.adj |
|---|---|---|---|---|
| Basal vs CLDNlow | 5447 | 0.76[0.75-0.77] | 2.23E-263 | 2.67E-262 |
| ERBB2 vs CLDNlow | 5447 | 0.71[0.71-0.72] | 0.00E+00 | 0.00E+00 |
| LumA vs CLDNlow | 5447 | 0.72[0.72-0.73] | 0.00E+00 | 0.00E+00 |
| LumB vs CLDNlow | 5447 | 0.65[0.64-0.66] | 0.00E+00 | 0.00E+00 |
| Normal vs CLDNlow | 5447 | 0.82[0.81-0.83] | 1.77E-103 | 1.60E-102 |
