# Supplementary figures and images for: Claudin-low breast cancers: clinical, pathological, molecular and prognostic characterization
Source: Mol Cancer. 2014 Oct 2;13:228. doi: 10.1186/1476-4598-13-228 (PMC4197217; doi:10.1186/1476-4598-13-228)

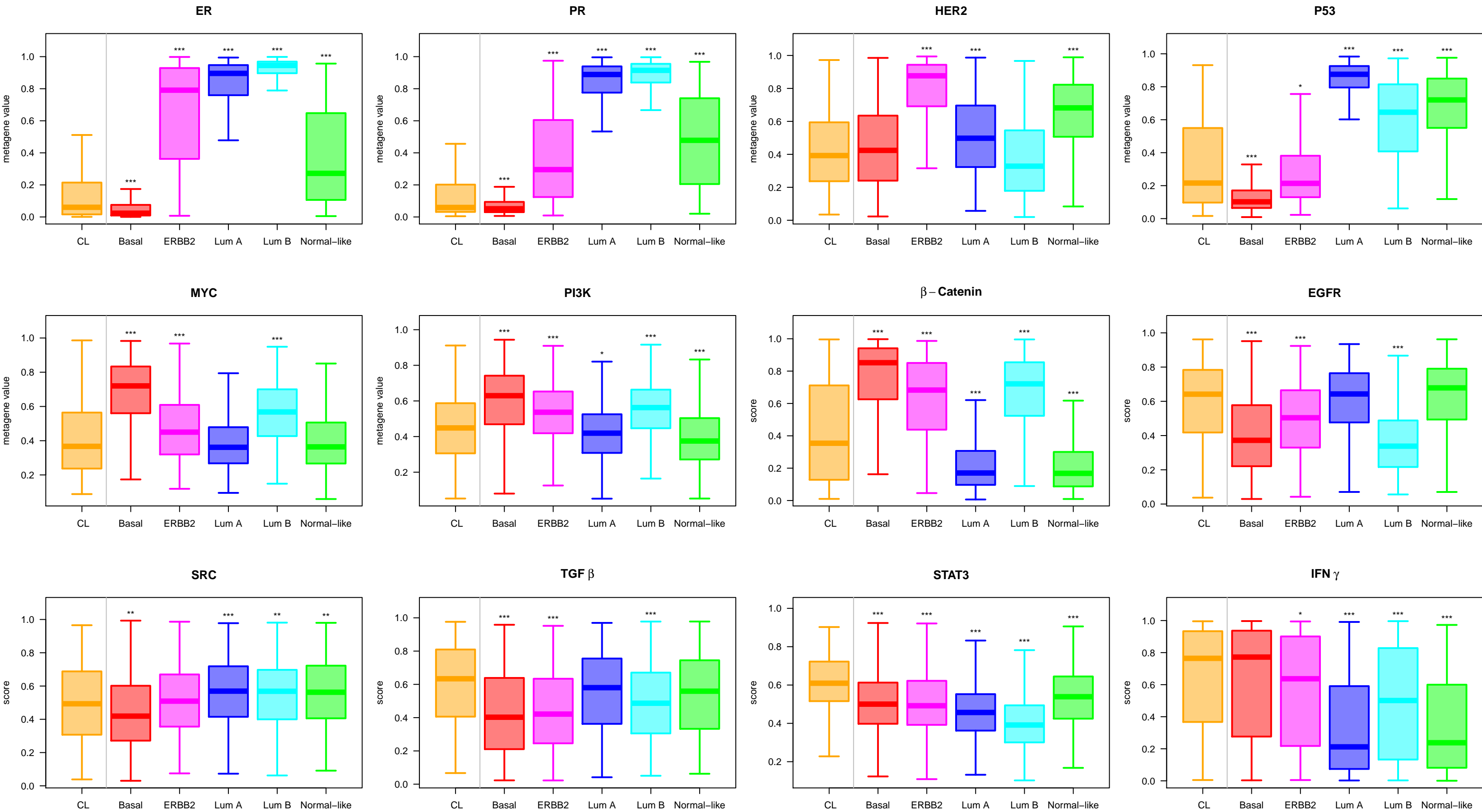

Supplement: Supplementary file 4 — Additional file 4: Figure S1: Comparison of probabilities of pathway activation across molecular subtypes. Box plots of probabilities of activation of 12 pathways from [44]. P-values (t-test) of comparisons between CL and each of the other subtypes are shown as follows: *, ≤5%; **, ≤1%; ***, ≤0.1%. (PDF 11 KB) [file 12943_2014_1429_MOESM4_ESM.pdf]

*CDH1*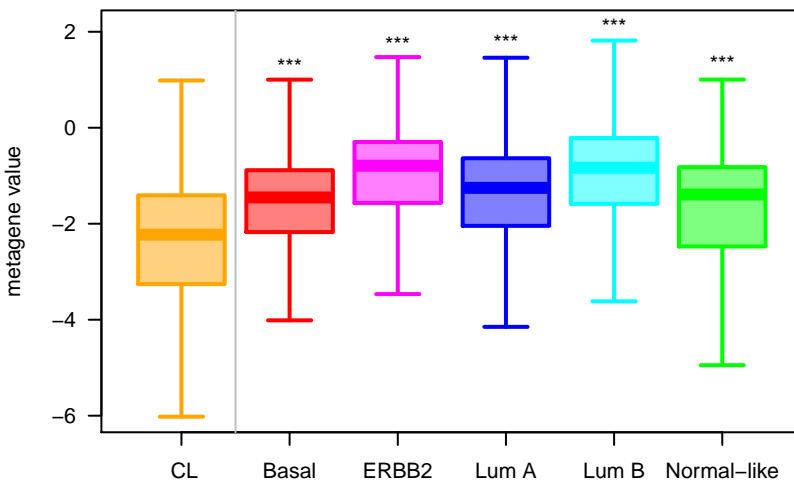*CLDN3*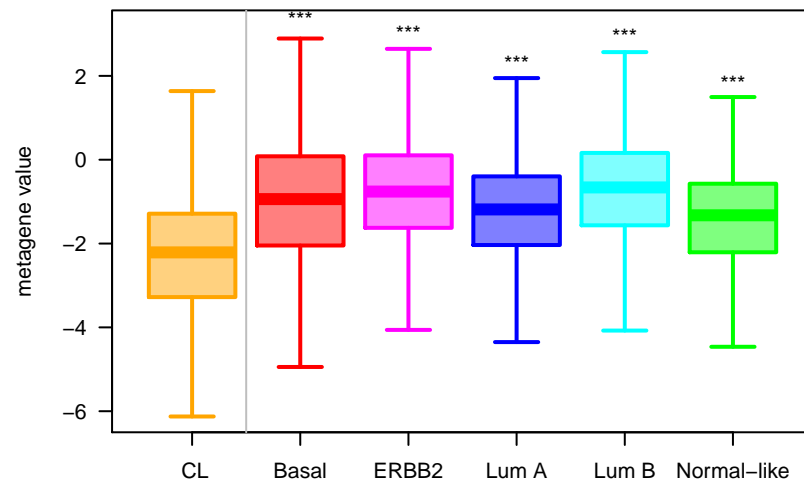*CLDN4*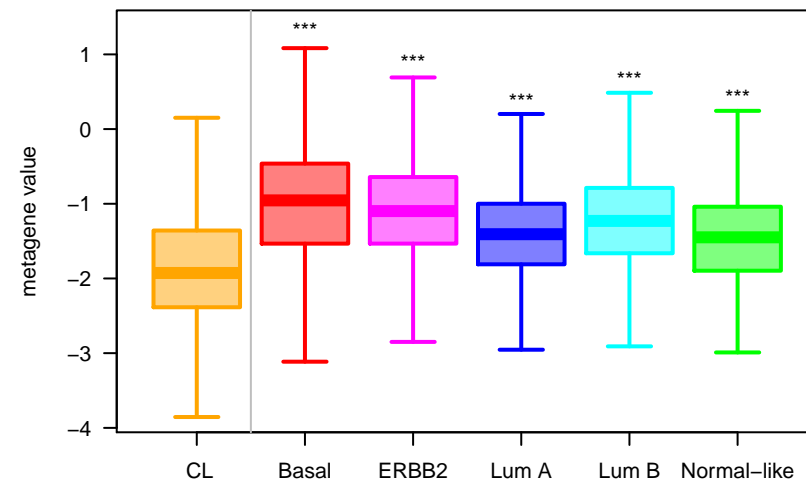*CLDN7*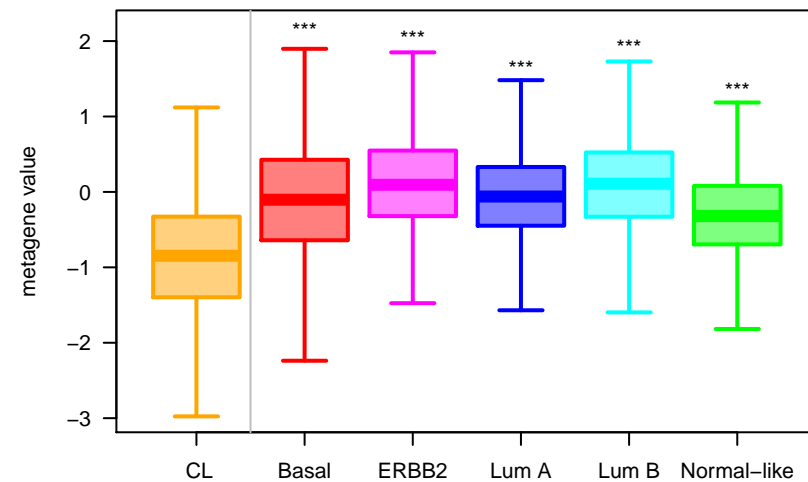*OCLN*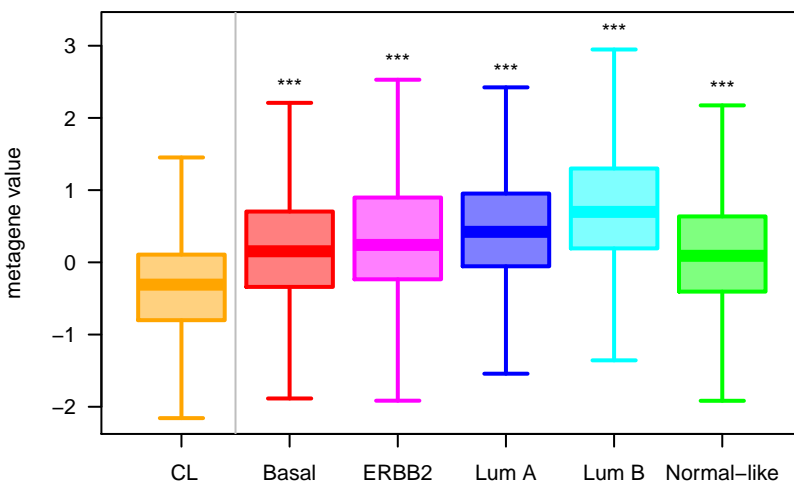*VIM*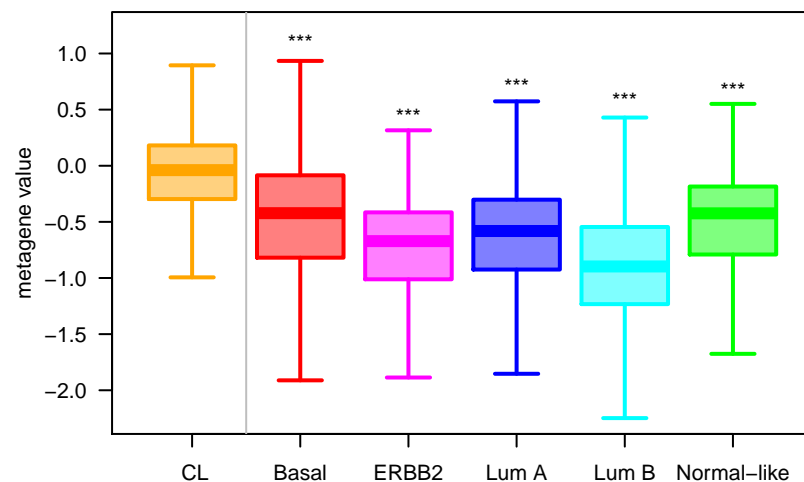*SNAI1*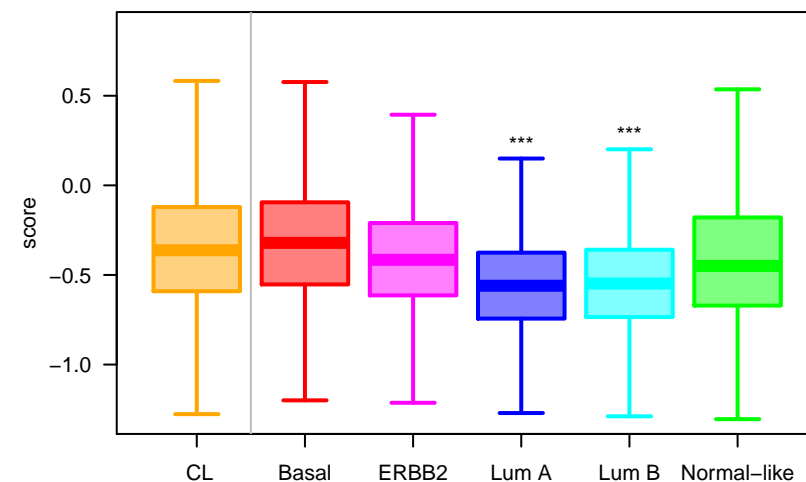*SNAI2*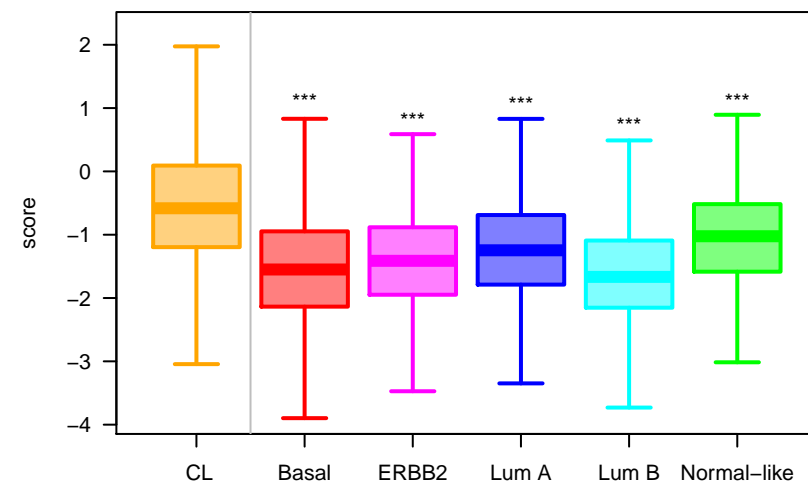*TWIST1*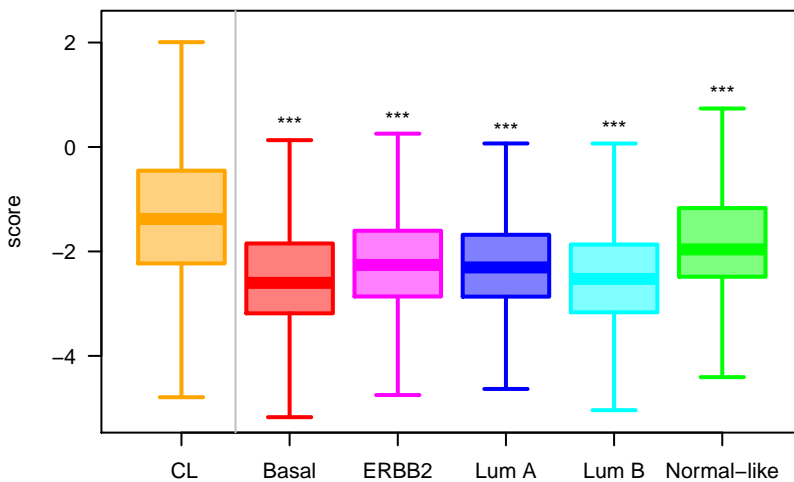*TWIST2*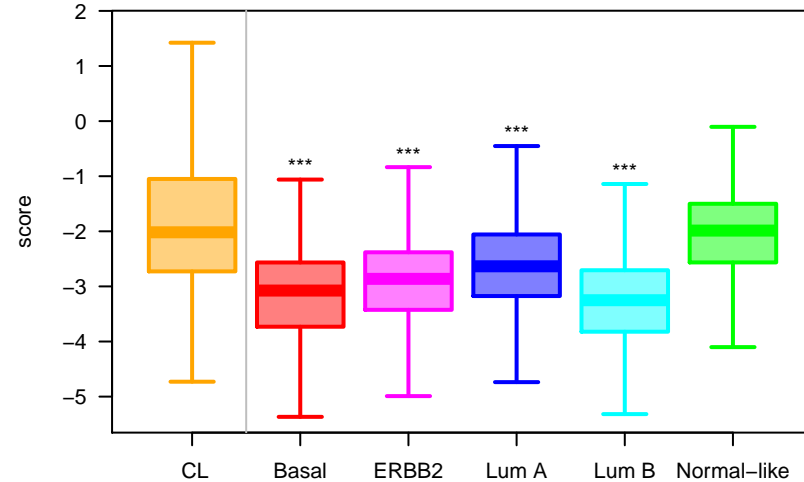*ZEB1*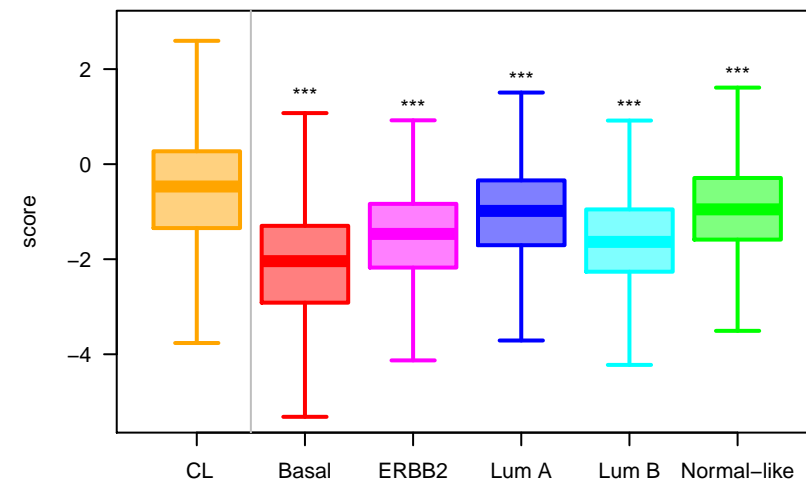*ZEB2*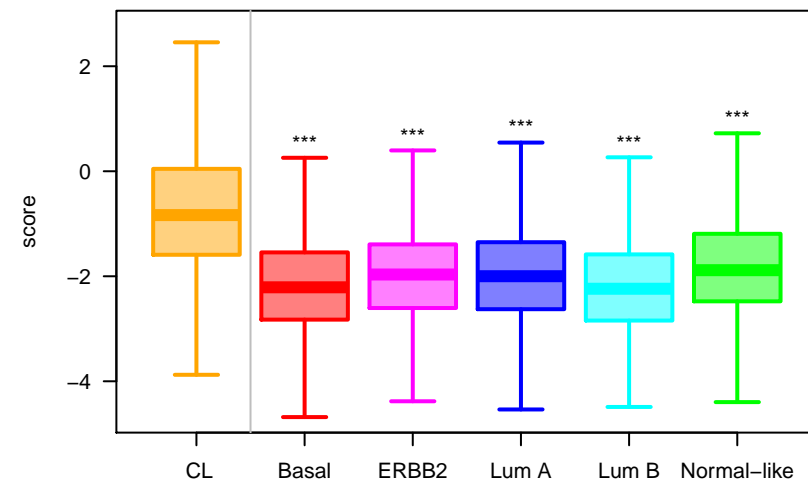

Supplement: Supplementary file 6 — Additional file 6: Figure S2: Comparison of mRNA expression levels of genes associated with EMT across molecular subtypes. Expression values are log2-scaled. P-values (t-test) of comparisons between CL and each of the other subtypes are shown as follows: *, ≤5%; **, ≤1%; ***, ≤0.1%. (PDF 11 KB) [file 12943_2014_1429_MOESM6_ESM.pdf]
